# PEF-Assisted Extraction of Proteins and Bioactive Compounds from Duckweed (*Lemna* ssp.): An Exploratory Proof-of-Concept Study

**DOI:** 10.3390/foods15152671

**Published:** 2026-07-29

**Authors:** Patricia Maag, Jörg Schäffer, Sabine Grüner, Cornelia Rauh, Özlem Özmutlu Karslioglu

**Affiliations:** 1 Institute of Food Technology, Weihenstephan-Triesdorf University of Applied Sciences, Am Staudengarten 11, 85354 Freising, Germany; oezlem.oezmutlu@hswt.de; 2 Food Biotechnology and Food Process Engineering, Technical University of Berlin, Königin-Luise-Straße 22, 14195 Berlin, Germany; cornelia.rauh@tu-berlin.de; 3 Department of Bioengineering Sciences, Weihenstephan-Triesdorf University of Applied Sciences, Am Hofgarten 10, 85354 Freising, Germany; joerg.schaeffer@hswt.de (J.S.); sabine.gruener@hswt.de (S.G.)

**Keywords:** nonthermal processing, duckweed valorization, cell permeabilization, protein recovery, polyphenols, green processing

## Abstract

Duckweeds (*Lemna minor*, LM and *Lemna gibba*, LG) represent promising protein-rich biomass for sustainable food ingredients, yet efficient physical aqueous extraction from fresh material remains largely unexplored. This proof-of-concept study presents the first application of pulsed electric fields (PEF) pre-treatment to enhance protein and bioactive compound release from homogenized *Lemna* ssp. suspensions. PEF parameters (1–8 kV cm^−1^, unipolar) and homogenization intensity (10 s vs. 5 min Ultra Turrax) were screened by quantifying protein yields, cell disintegration index (Z_d_), energy input and chlorophylls, carotenoids and polyphenols. Homogenization proved essential, with 5 min treatment alone yielding 22.0–34.6% protein extraction; subsequent 5 kV cm^−1^ PEF increased this by 11.0–16.8% (33% LG; 51% LM). Among the tested conditions, 5 kV cm^−1^ following 5 min homogenization maximized yields; however, it required the highest energy input (~239 kJ/kg), while 8 kV cm^−1^ after 10 s offered a promising efficiency trade-off (~54 kJ/kg). Z_d_ values ≥ 0.9 confirmed electroporation as the mechanism, with PEF and homogenization ~5–25× more energy efficient than the freeze–thaw process. Bioactive compounds nearly doubled under optimal conditions, with trends mirroring protein release. These findings validate PEF as a green pre-treatment strategy for the protein recovery of *Lemna* spp., while also offering mechanistic understanding that can support its future implementation in extraction processes.

## 1. Introduction

Duckweeds are high in protein (20–>35%) and rich in valuable compounds such as carotenoids, chlorophyll, polyphenols and omega-3-fatty acids [1,2,3,4]. Consequently, duckweeds gained increasing attention as a sustainable alternative protein source for food and feed applications, highlighting their comparatively rapid proliferation, and superior protein productivity per unit cultivation area [5,6]. For example, open-pond cultivation in Thailand achieved annual protein yields of approximately 2080 kg ha^−1^, substantially exceeding those of soybean (303 kg ha^−1^), nuts (229 kg ha^−1^) and maize (179 kg ha^−1^) [7]. Moreover, an advanced controlled and automated indoor vertical farming system produced 0.9 kg fresh biomass day^−1^ (53 g dry biomass, 32% protein), corresponding to 620 kg ha^−1^ month^−1^ (7.54 t ha^−1^ year^−1^ dry matter) [8], comparable to the biomass productivity reported by Chakrabarti, R. et al. 2018 (702.5 kg ha^−1^ month^−1^) [9]. Against this background, duckweed has attracted growing interest as an alternative protein source [10,11,12], particularly regarding the development of advanced protein extraction strategies, including enzymatic, fermentation-based and innovative technological approaches to enhance its utilization as a dietary ingredient [13]. Recent findings demonstrated that duckweed protein concentrate can enhance textural properties of food products, such as crispness, in vegan products, e.g., baked goods and snack bars [14]. The sustainable recovery of high-quality proteins and other valuable nutritional compounds has emerged as a key priority in the food industry and is growing in importance in light of rising consumer demand. In this context, economically viable non-thermal extraction technologies offer significant potential by reducing chemical requirements and processing times while maintaining the quality of the recovered biomolecules [12,15,16]. For instance, ultrasound-assisted extraction has already been shown to improve protein yields, amino acid profiles or protein digestibility in duckweed species [2,17,18]. Furthermore, pulsed electric fields (PEF) represent an innovative, non-thermal technology characterized by rapid processing and enhanced extraction efficiency. Although PEF has not yet been applied to duckweed protein extraction [11], it is considered a promising approach for facilitating the release of proteins and bioactive compounds from plant cell membranes [5]. In other fields of food processing and research, pulsed electric field technology has already been investigated for various applications. It has been shown to be effective in preserving foods by achieving microbial and endospore inactivation, with particular success in liquid products such as fruit juices and other beverages and thereby attracting considerable interest due to its non-thermal nature to the food industry [19]. In addition, its application can soften plant tissues, e.g., in potatoes and beetroot, leading to reduced frying or cooking times and supporting the retention of color and nutritional quality [19,20,21]. Furthermore, in research, PEF technology has demonstrated high efficiency as an extraction technique by inducing a breakdown of cell membranes due to electroporation, which forms reversible or irreversible pores in plant cell membranes [22]. Low electric field strengths of 0.5 to 5 kV cm^−1^ are already sufficient to cause irreversible permeabilization (electroporation) of plant cells, leading to the intracellular release of valuable compounds, e.g., proteins, lipids, bioactive substances, minerals [23,24,25,26]. Moreover, extraction efficiency is influenced by the number of pulses, pulse width and pulse type [23]. In studies focusing on the intracellular release of compounds from plant materials, particularly algae, unipolar (monopolar) pulses were frequently employed as well as bipolar pulses, both showing enhanced electroporation efficiency and improved extraction yields [25,27,28,29,30,31,32,33]. PEF-assisted extraction of secondary metabolites, proteins and oils from promising plant sources has gained significant attention. For example, extensive research has already been successfully conducted on microalgae, focusing on the extraction of nutrients, such as proteins and carbohydrates, particularly from *Chlorella vulgaris* [27,33,34,35], as well as antioxidants and minerals [36], or C-phycocyanin from *Arthrospira platensis* (Spirulina) [29,37,38]. Given the demonstrated efficacy of PEF technology as an innovative, non-thermal extraction approach, particularly its capacity to operate with minimal or no chemical solvent input, further comprehensive investigations are required to evaluate its applicability and efficiency for protein extraction from protein-rich plant matrices. 

This study presents the first exploratory screening of pulsed electric field (PEF)-assisted extraction applied to the duckweed species *Lemna minor* (LM) and *Lemna gibba* (LG). It aims to assess the effectiveness of PEF as a cell disruption pre-treatment to enhance the release of intracellular proteins and bioactive constituents from Lemna spp. biomass. The approach is evaluated in the context of integration into subsequent downstream processing steps, which may include protein purification and concentration. Although the primary objective is protein recovery for food applications, the co-extraction of valuable bioactive compounds such as chlorophylls, carotenoids and polyphenols represents additional potential benefits.

## 2. Materials and Methods

### 2.1. Duckweed and Cultivation Conditions

*Lemna gibba* (LG), Clone No. 7796 and *Lemna minor* (LM), Clone No. 9441, were originally obtained from the University of Jena, Germany. The duckweed species are cultivated in a greenhouse at the Institute of Horticulture, Weihenstephan-Triesdorf University of Applied Sciences, using natural light in summer and supplemental light in winter at controlled temperatures of around 20 ± 2 °C. Supplemental lighting with a high-pressure sodium lamp (HPS) (Philips SON-T Agro 400W, Philips, Milan, Italy) was activated between 6 am and 10 pm when the outside photosynthetically active radiation (PAR) felt below 300 µmol m^−2^ s^−1^.

The duckweed was harvested and washed according to a defined SOP (standard operating procedure) in the order of manual harvesting with a conventional kitchen sieve, washing three times with tap water, while deionized water was used for the last washing step to avoid the influence of salts and minerals during the PEF process. Subsequently, centrifugation was performed using a conventional salad spinner. The dry matter of the fresh biomass in percentage (%) was 5.10 ± 0.98 for LM and 4.54 ± 0.55 for LG and served as the starting point for calculating the target biomass of 1 wt% used for the experiments.

### 2.2. PEF-Assisted Extraction

**Figure 1:**
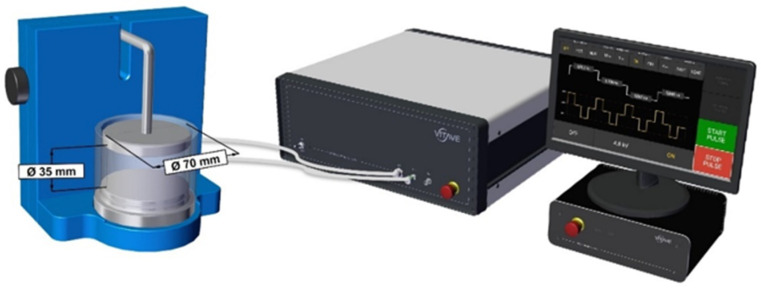
OmniPEF LAB generator with touch-display and high-voltag generator (r.) linked to a batch-treatment chamber (l.) (The exemplary illustration was provided by Vitave Tech, Prague, Czech Republic and was slightly adapted).

The duckweed biomass was pulsed in batch treatment using the model OmniPEF LAB (Figure 1, treatment chamber is shown disproportionately larger for a better view) from Vitave Tech (Prague, Czech Republic). The electric field strengths (*E* in kV cm^−1^) applied to each sample were 1, 3, 5 and 8 kV cm^−1^, which result from the voltage (*U* in kV) applied and the distance between the two electrodes (*d* in cm), according to Equation (1). During PEF treatment, the average current was 45.3 ± 10.4 A, 136.5 ± 24.9 A, 216.8 ± 10.5 A, and 424.4 ± 40.0 A at electric field strengths of 1, 3, 5 and 8 kV cm^−1^, respectively. Moreover, the temperature increase (ΔT) following PEF treatment was minimal, ranging from 1.1–2.9 °C across the applied electric field strengths (1–8 kV cm^−1^), with the initial suspension temperature maintained at approximately 20 °C. The desired electric field strengths were achieved primarily by varying the electrode gap distance and where required, by adjusting the applied voltage. Furthermore, unipolar (monopolar) pulses were applied as a train of 100 pulses with a constant pulse width of 5 µs, while the pulse pause was kept constant at 5 µs, resulting in a total processing time of 1000 µs.

(1) $$E=\frac{U}{d}$$

**Figure 2:**
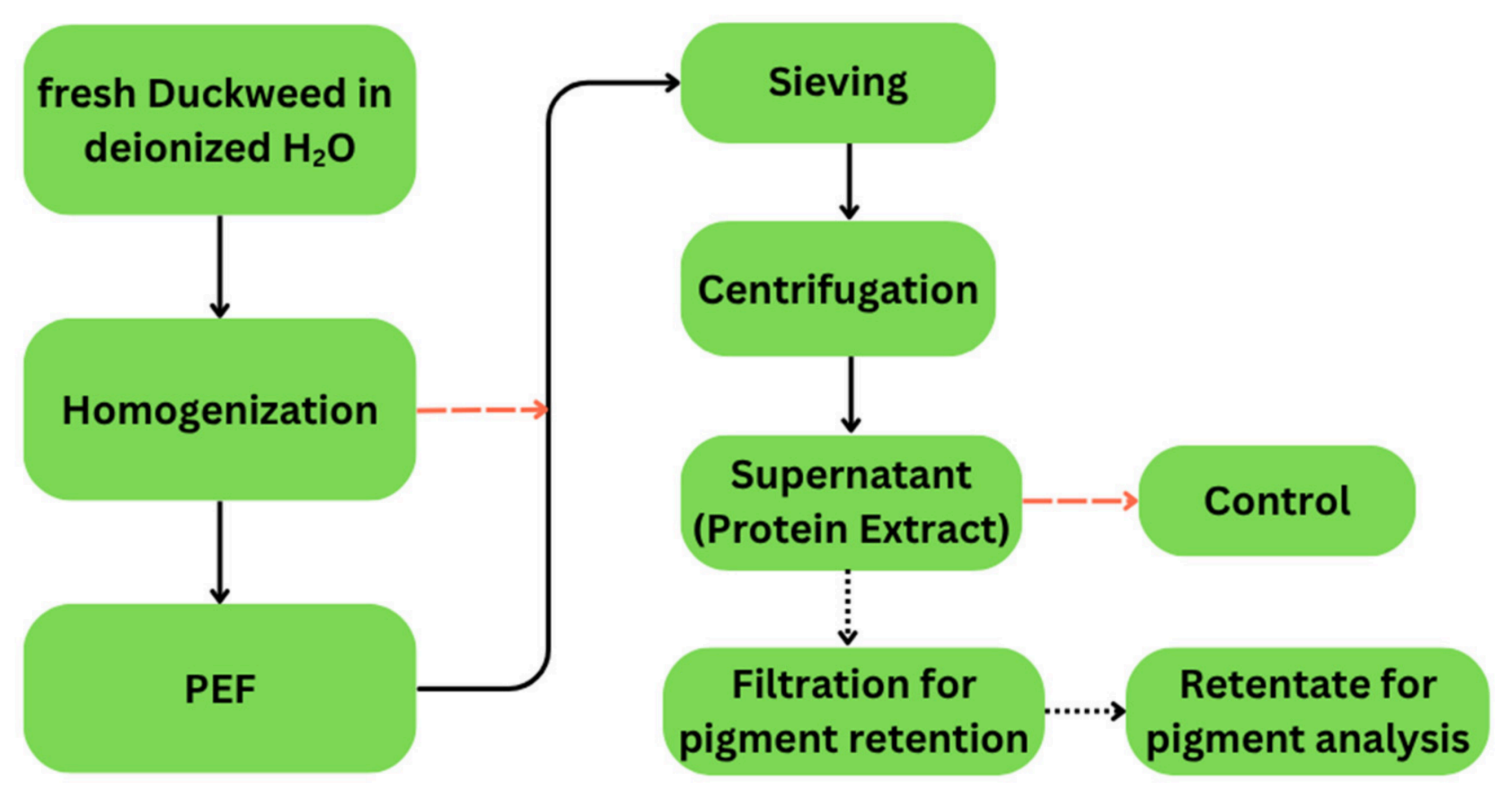
PEF-assisted extraction process of *Lemna* ssp.

The PEF process is illustrated in Figure 2. Prior to PEF treatment, duckweed was dispersed in distilled water at 1% (*w*/*w*, dry matter basis), corresponding to a dry matter-to-water ratio of 1:99 (*w*/*w*), by weighing an amount equivalent to 1.0 g dry matter and adding distilled water to obtain a total suspension mass of 100 g. An aqueous medium was selected to evaluate the extraction efficiency resulting exclusively from the PEF treatment. To investigate the influence of PEF-assisted protein extraction, prior mechanical disruption of the plant material was necessary. Accordingly, two levels of homogenization intensities were evaluated, using an ULTRA-TURRAX from MICCRA, Buggingen, Germany (Model D-9, Serial No. 4004) at 11,000 rpm, both in combination with PEF (Figure 2, solid black arrow) and as controls without PEF (orange dashed arrow). Mild homogenization was performed for 10 s, whereas high-intensity homogenization was conducted for 5 min. The latter represents the maximum homogenization time beyond which no further increase in conductivity was observed (see Section 3.3). Additionally, a third control sample, subjected to three freeze–thaw cycles was included to represent a conventional processing condition for comparison. Following PEF treatment, the suspension was transferred into the batch treatment chamber and the respective PEF parameters were applied. Subsequently, the treated sample was subjected to sieving (36–38 µm mesh size) and centrifugation at 4600 rcf for 20 min at room temperature. The obtained supernatant was transferred to an airtight container and stored at −25 °C until further analysis of proteins, polyphenols, chlorophylls and carotenoids, following pigment filtration (Figure 2, black dotted arrow) with a glass fiber filter (Whatman GF/F, 90 mm diameter, 0.7 µm particle retention, Cytiva, Amersham, UK) and analysis, which is explained in Section 2.6 and Section 2.7. In this manuscript, the term extraction refers to the release of compounds such as proteins or secondary metabolites (chlorophylls, carotenoids and polyphenols) into an aqueous medium by PEF-assisted extraction.

### 2.3. Protein Measurement

The content of soluble proteins in the supernatant was analyzed with the BCA-protein assay according to the protocol of Thermo Fisher Scientific TM Pierce (Waltham, MA, USA) and based on the original description by Smith, P.K., et al. 1985 [39] with minor modification, for which the Bovine-serum albumin (BSA) was used as calibration standard. The BSA calibration curve, ranging from 0 to 2000 µg/mL, was prepared according to the manufacturer’s protocol with additional concentration points included to enable more detailed quantification (see Supplementary Material S1, Figure S1). The results were expressed as mg equivalent of BSA per g of dry biomass and presented in percentage after relating it to the total protein content of duckweed flour (DF, Equation (2)), determined by the Dumas method (Nx5.8). For each sample, three independent preparations were made, and each was measured in triplicate.

(2) $$Y_{proteins\;in\;\%}=\frac{{Protein}_{BCA}}{{Protein}_{total\;DF}}\times 100$$

### 2.4. Energy Input

Determination of the total specific energy input (W_spec_) in kJ/kg requires the calculation of the pulse energy (W_pulse_, kJ) in advance using Equation (3), where U is the voltage in kV, I is the current in Ampere and τ is the pulse width in µs, converted to seconds (* 10^−6^). The total specific energy input (W_spec_) was calculated using the number of pulses (n) multiplied by W_pulse_ and divided by the total mass (m) of the duckweed suspension in distilled water obtained in g and converted to kg (* 10^−3^), shown in Equation (4). Energy inputs of homogenization (W_H_) using the ULTRA-TURRAX (UT) from MICCRA, Buggingen, Germany (Model D-9, Serial No. 4004) with W_UT_ in kJ (Equation (5)) and from freeze–thaw treatment (Q_Total_, Equation (9)) were calculated based on the specific heat capacity of water (Q_1_, c_water_ = 4.18 kJ/kg·K) and ice (Q_3_, c_ice_ = 2.1 kJ/kg·K) and ΔT for the difference in temperature as well as the melting enthalpy of water (Q_2_, q_melt_ = 333.9 kJ), according to Langeheinecke, K., et al. 2003 [40]. These values (Equations (6)–(9)) were incorporated into the total specific energy input.

PEF

(3) $$\mathrm{Pulse}\;\mathrm{energy}\;W_{pulse}=U*I*\tau$$

(4) $$\mathrm{Total}\;\mathrm{specific}\;\mathrm{energy}\;W_{spec}=\frac{{n*W}_{pulse}}{m}$$

(5) $$\mathrm{Homogenization}\;W_{H}=\frac{W_{UT}}{m}$$

Freeze–Thaw

(6) $$1.\mathrm{Cooling}+20\;\mathrm{to}\;0\;{}^{\circ}C\;Q_{1}=m*c_{water}*\Delta T$$

(7) $$2.\mathrm{Freezing}\;\mathrm{at}\;0\;{}^{\circ}C\;Q_{2}=m*\;q_{melt}$$

(8) $$3.\mathrm{Freezing}\;0\;\mathrm{to}-20\;{}^{\circ}C\;Q_{3}=m*c_{ice}*\Delta T$$

(9) $$\mathrm{Total}\;\mathrm{energy}\;{\mathrm{input}}_{\mathrm{freeze-thaw}}\;Q_{Total}=Q_{1}+Q_{2}+Q_{3}$$

### 2.5. Cell Disintegration

The cell disintegration index (Z_d_) indicates the degree of cell damage or permeabilization in plant tissue and was calculated based on the electrical conductivity measurement of both intact and PEF-treated plant tissue in relation to completely damaged plant tissue. The electrical conductivity was measured (conductivity- and pH-meter, Model 1100 H, accuracy ≤ 0.005 pH ± 1 digit, VWR, Ismaning, Germany) before and after PEF treatment. The conductivity value for completely damaged plant tissue, for each *Lemna* ssp. was determined by homogenization at 11,000 rpm using the ULTRA-TURRAX (UT) from MICCRA, Buggingen, Germany (Model D-9, Serial No. 4004), considering a temperature increase of 2.4 °C ± 0.91, in combination with PEF. The highest achieved conductivity levels, without significant difference, were at 8 kV cm^−1^ following 10 s UT and 5 kV cm^−1^ following 5 min UT for *Lemna minor* with the latter also applying for *Lemna gibba*. The cell disintegration index (Z_d_) was determined using Equation (10) [31,37], where the conductivity value of D_i_ represents intact cells, D_PEF_ denotes PEF-treated cells and D_d_ corresponds to completely damaged cells.

(10) $$Z_{d}=\frac{D_{PEF}-D_{i}}{D_{d}-D_{i}}\;$$

### 2.6. Total Chlorophyll and Carotenoids

Chlorophyll (Chl) *a* and *b* were extracted using a double extraction method based on existing protocols [41,42]. The calculation coefficients for Chl *a* and *b* were applied according to Ritchie, R.J., 2006 [43]. After PEF extraction and centrifugation, the supernatant was filtered through a fiber glass filter to capture the chlorophylls and carotenoids. Then, the fiber glass filter was placed in a 15 mL centrifuge tube and a spatula tip of ~ 4 mg MgCO_3_ (Sigma-Aldrich Chemie GmbH, Darmstadt, Germany) and 6 mL of ethanol ≥ 96% (Carl Roth GmbH + Co. KG, denatured, Karlsruhe, Germany) was added and mixed by stirring for 10 min at 350 rpm. Subsequently, the suspension was incubated in a water bath at 80 °C for 5 min. During incubation, centrifuge tubes were sealed with Parafilm to prevent ethanol evaporation while allowing gas release. The subsequent steps were performed as described in a previous publication [2].

Absorbance for chlorophyll *a* and *b* was measured at 649 nm and 665 nm, respectively, and at 750 nm to correct for background interference caused by the intrinsic color of the samples. Total carotenoids were quantified by measuring absorbance at 470 nm using a spectrophotometer SPECORD PLUS 210, Jena, Germany. The calculation of the Chl a and b content is provided in Equations (11) and (12), expressed in mg per g sample based on dry weight. To calculate Chl *a* and *b*, the absorption values (Ax), dilution factor (DF), the solvent volume (S) in mL and sample weight (W) in g are required. The total Chl *a* + *b* content was calculated as the sum of the results from Equations (11) and (12) [41,43]. The total carotenoid content (C) was calculated according to Equation (13) based on the method described by Lichtenthaler, K. and Wellburn, A.R., 1983 [44] using 96% (*v*/*v*) ethanol as the solvent. In this context, $C_{ch}^{a}$ and $C_{ch}^{b}$ represent the concentrations (mg of pigment/g dry weight (DW)) of chlorophyll a and chlorophyll b, respectively.

(11) $$Chl\;a\;\left[mg/g\right]=\frac{\left(13.5275*{(A}_{665}-A_{750}\right)-5.2007*\left(A_{649}-A_{750}\right))*DF*S}{W}$$

(12) $$Chl\;b\;\left[mg/g\right]=\frac{\left(22.4327*{(A}_{649}-A_{750}\right)-7.0741*\left(A_{665}-A_{750}\right))*DF*S}{W}$$

(13) $$C=\frac{\left({1000A}_{470}-2.05C_{ch}^{a}-114.8C_{ch}^{b}\right)}{245}$$

#### 2.6.1. UV–Vis Spectra of Chlorophyll and Carotenoids

UV–Vis spectrograms were performed using a Specord-Plus 210 (Analytik Jena, Germany) double-beam spectrophotometer with semi-microcuvettes. The measuring range was selected from 350 nm to 800 nm with a slit width of 1 nm and a data interval of 0.5 nm. The integration was set to 250 ms, corresponding to a scan speed of 2 nm/s. The PEF-treated samples were filtered through a glass fiber filter (Whatman GF/F, 90 mm diameter, 0.7 µm particle retention). The retentate, along with the filter, was transferred to a centrifuge tube and extracted in 96% (*v*/*v*) ethanol, following the extraction protocol described in Section 2.6. The resulting supernatant was subsequently used for pigment analysis. Multiple baseline-corrected spectra were recorded and averaged, accounting for sample dilution. A 95% confidence interval was calculated for selected values.

#### 2.6.2. Thin-Layer Chromatography

Thin-layer chromatography (TLC) was conducted on 0.5 mm silica gel aluminium sheets (Hager and Meyer-Bertenrath, length: 200 mm) [45]. Samples were applied using a piston pipette, with a maximum loading volume of 10 µL per application; multiple applications were performed as needed. The mobile phase consisted of hexane–isopropanol–water in a ratio of 100:10:0.25. Chromatographic separation was carried out immediately after drying the applied spots, using a closed glass chamber pre-saturated with the mobile phase vapor. The ascending phase of chromatography was halted once the distance between the solvent front and the upper edge of the silica gel layer reached approximately 5–10 mm (after ~60 min). Immediately upon removal of the plate, the solvent front was marked with a graphite pencil. After drying, the TLC plate was digitized using a flatbed scanner (EPSON), and image contrast was enhanced using GIMP software 2.10 by adjusting the black point from 0 to 235.

### 2.7. Polyphenols

The Folin–Ciocalteu (FC) method was applied as described by Singleton, V. L.; Orthofer, R., 1999 [46] with minor modifications. The total phenolic content was expressed as mg gallic acid equivalent (mg GAE/mL) (97.5–102.5% titration, Sigma-Aldrich, Germany) using a standard calibration curve (R^2^ = 0.9995, see Supplementary Materials S7, Figure S13), which was prepared from 0.005–0.07 mg mL^−1^ [47]. The FC-reagent was diluted 1:10 with methanol 6% (>99.8%, ultrapure, spectrophotometric grade, Thermo Fisher Scientific, Waltham, MA, USA) and wrapped in aluminum foil. To initiate the reaction, 120 µL PEF-extract was mixed with 900 µL FC-reagent (Merck KGaA, Darmstadt, Germany) in a centrifuge tube (2 mL, plastic, sterile) and incubated at room temperature in the dark for 5 min. To optimize the reaction, 900 µL Na_2_CO_3_ solution (6%) (Merck KGaA, Darmstadt, Germany) was added to adjust the pH to alkaline conditions (pH 10), followed by a 90 min incubation at room temperature in the dark. The absorption was measured at 760 nm [46,48].

### 2.8. Statistical Analysis

Statistical analyses were performed based on appropriate procedures and methods using Numiqo Statistics Calulator (numiqo Team, 2026, https://numiqo.com/), a web-based statistical analysis platform. This includes standard procedures in the order of the test of normal distribution (Kolmogorov–Smirnov Test). The results were then statistically analyzed using one-way analysis of variance (ANOVA) at a *p* < 0.05 significance level, followed by a Tukey post hoc test to determine statistical significance of the data collected.

## 3. Results and Discussion

### 3.1. PEF-Assisted Protein Extraction

The application of PEF-assisted protein extraction effectively facilitated the release of proteins from plant cell structures into the surrounding aqueous medium at specific electric field strengths, with further improvements observed when suitable homogenization parameters were applied prior to treatment. Furthermore, preliminary scientific work has compared and evaluated the pulse types (unipolar, bipolar) as well as different incubation periods following PEF treatment (see Supplementary Materials S2, Figure S2). Although incubation time has been identified as a relevant factor influencing protein release, e.g., in microalgae [36], the same could not be confirmed for *Lemna* spp. within a comparable incubation range of 0–120 min after PEF treatment. Additionally, variation in pulse type (unipolar, bipolar) did not result in statistically significant differences in extraction efficiency. However, at higher electric field strengths, most notably at 5 kV cm^−1^, a trend toward increased protein release was observed for unipolar pulses. These results are also in agreement with observations reported by Carullo, D. et al., 2020 [30] and Kronbauer, M. et al., 2023 [25], who applied a unipolar pulse mode for plant protein extraction. Based on these findings, subsequent experiments were performed using unipolar pulse conditions, while incubation time was not considered as an experimental variable.

**Figure 3:**
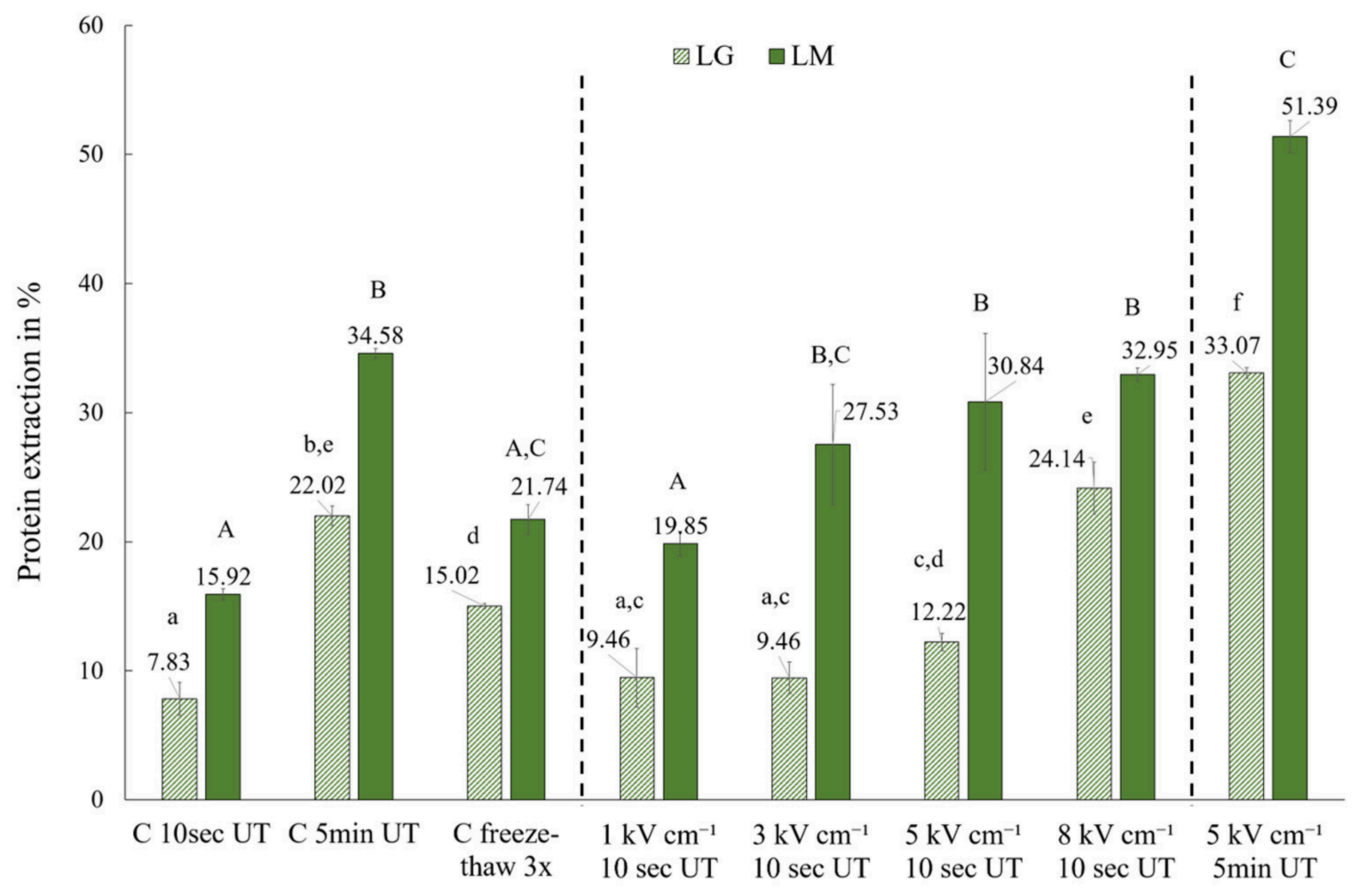
PEF-assisted protein extraction from *Lemna gibba* (LG) and *Lemna minor* (LM) with different lowercase letters for LG and uppercase letters for LM, showing significant difference at *p* < 0.05, ac-cording to Tukey post hoc test.

Distinct species-specific effects were observed for PEF-assisted extraction for the two *Lemna* species. LM showed a significant enhancement in protein release at 1 kV cm^−1^ (19.85%) compared to the untreated, homogenized control sample (C10secUT; 15.92%). This effect intensified with increasing electric field strength, reaching 32.95% at 8 kV cm^−1^, showing no significant difference between 5 and 8 kV cm^−1^ (Figure 3). In contrast, LG showed an increasing trend in protein release with increasing field strength; however, a statistically significant improvement over the control sample C10secUT was observed only at 8 kV cm^−1^.

The influence of PEF treatment on protein extraction efficiency differed between the two duckweed species. LM showed higher protein extraction yields than LG. However, direct structural and morphological comparisons that could explain the observed differences between *Lemna minor* (Cl. 9441) and *Lemna gibba* (Cl. 7796) are currently lacking in the literature. To date, only a small number of botanical studies have provided separate morphological characterizations of the individual species, from which no clear explanations can be derived regarding the difference in extractable protein content [49,50,51,52,53]. Moreover, the examined protein contents for LM or LG flour vary based on the literature results, for example, values of 32–45% for LM, but lower protein values for LG (20–30%) are confirmed [13,52,54]. However, cultivation parameters and environmental impacts (e.g., temperature, light source (natural, artificial) and exposure time, nutrient solution) can lead to protein fluctuations, as protein contents below 30% [1] were also reported for LM as well as higher protein contents of 33–36% for LG [2,55].

**Figure 4:**
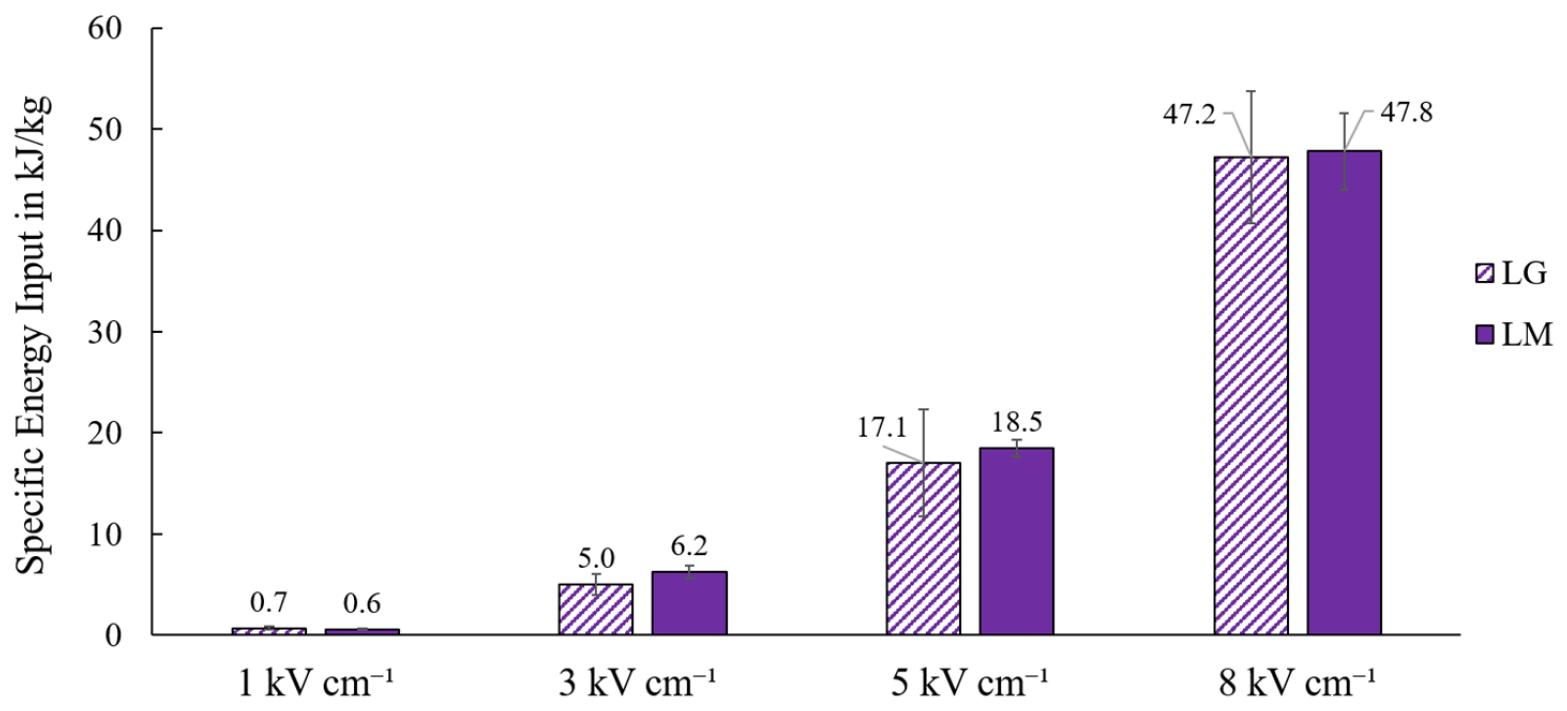
Specific energy input in kJ/kg from PEF treatment.

Furthermore, in this study, a freeze–thaw control sample was used as a benchmark for comparison to the conventional cell disruption method. This proved to be even more effective in terms of cellular disintegration, resulting in higher protein release compared to the LM control sample C10secUT, while also outperforming its subsequent PEF treatment at 1 and 3 kV cm^−1^ for LM and even at 5 kV cm^−1^ for LG. However, PEF treatment of LM at 5 kV cm^−1^ or higher yielded a significantly higher protein extraction yield than the corresponding freeze–thaw sample. As prior cell disruption strongly influences the overall protein release during PEF-assisted extraction, an extended homogenization time of 5 min was investigated to establish a new baseline condition. The homogenization time of 5 min represents the maximum homogenization time beyond which no further increase in conductivity was observed after elongated mechanical disruption using the ULTRA-TURRAX (see Supplementary Materials S3, Figure S3). Homogenization alone for 5 min resulted in a substantial increase in protein extraction in the control samples, reaching 34.58% for LM and 22.02% for LG. These values were equal to or exceeded the extraction efficiencies obtained from samples subjected to 10 s of homogenization followed by PEF-assisted extraction. Since no significant difference in protein extraction was observed for the LM samples at field strengths between 5 and 8 kV cm^−1^, the significantly lower specific energy input at 5 kV cm^−1^ was used as the deciding factor for proceeding with further process steps (see Section 3.2, Figure 4). The application of a field strength of 5 kV cm^−1^ was therefore followed by a 5 min homogenization to investigate whether PEF-assisted protein extraction leads to a further increase in protein extraction. The results confirmed a significant increase in protein extraction of 11% for LG and around 17% for LM, relative to the respective 5 min homogenization controls. Based on these findings, the optimal conditions for PEF-assisted protein extraction were identified as 5 min of homogenization combined with an electric field strength of 5 kV cm^−1^ for both *Lemna* spp., yielding total protein extraction efficiencies of 33.07% for LG and 51.39% for LM, which means, e.g., for LM, an increase in extraction of approximately 50% of the total crude protein content (33.74% ± 0.06) into the aqueous phase.

It can be stated that homogenization duration prior to PEF-assisted protein extraction can be a crucial factor influencing protein extraction yields from plant cells. The relevance of the plant material disruption, like homogenization or pre-grinding, before PEF treatment, is also emphasized by Kronbauer, M. et al., 2023 [25], who demonstrated that PEF-assisted protein extraction from ground nettle leaves was considerably more efficient than from unprocessed leaves, achieving a maximum protein extraction yield of 68% at an electric field strength of 3 kV cm^−1^ (pulse width 40 µs, 2 Hz) in unipolar mode. Furthermore, Carullo, D. et al., 2021 [28] confirmed that homogenization under high shear stress significantly improved PEF extraction of water-soluble proteins and C-phycocyanin from *Arthrospira platensis*.

In contrast, a study on protein extraction from ground rapeseed leaves reported no improvement in extraction yield at a low electric field strength of 5 kV cm^−1^. A significant increase in protein yield, reaching up to ~80% in aqueous solution and outperforming ethanol extraction (25%), was only observed at 20 kV cm^−1^. The increased requirement for electric field strength was attributed to the presence of epicuticular wax on rapeseed leaves in the ground material, which likely acts as a barrier, hindering electroporation during PEF treatment [56,57]. The existing studies with PEF treatment on green leafy plants show that for plant-related structural characteristics of green leafy plants, including leaf morphology, intracellular composition as well as factors such as pre-crushing of plant tissue, the choice of extraction solvent and temperature applied during the extraction process can have a significant impact on the choice of PEF parameters in increasing extraction yields. This includes the total specific PEF energy input, encompassing parameters such as voltage, current, pulse width, number of pulses or frequency and pulse mode that play a decisive role and must be evaluated individually for each plant tissue [25,26,57]. Consequently, comparative analysis remains limited by the current lack of literature on PEF-assisted protein extraction in green biomass. Notably, the absolute absence of prior research evaluating *Lemna* spp. highlights a critical knowledge gap, underscoring the novelty of this work while defining a clear path for future optimization studies [5,11].

### 3.2. PEF Energy Input

The specific energy input, mainly derived from the pulse energy, and number of pulses on the duckweed biomass increased, as expected, with increasing electric field strength. This trend is consistent with the increased protein release observed under these conditions, as described in Section 3.1. Consequently, the highest specific energy input was recorded at 8 kV cm^−1^, as 47.2 kJ/kg for LG and 47.8 kJ/kg for LM. An energy input more than half as low, at 17.1 kJ/kg for LG and 18.5 kJ/kg for LM, was determined after PEF treatment at 5 kV cm^−1^ (Figure 4). These conditions also corresponded to the two highest levels of protein release for each *Lemna* ssp., illustrating the direct relationship between energy input and extraction efficiency. However, following a 10 s homogenization step, there is no significant difference in protein extraction yield between 5 and 8 kV cm^−1^. As energy consumption is significantly lower at 5 kV cm^−1^, this argues in favor of using this field strength.

**Figure 5:**
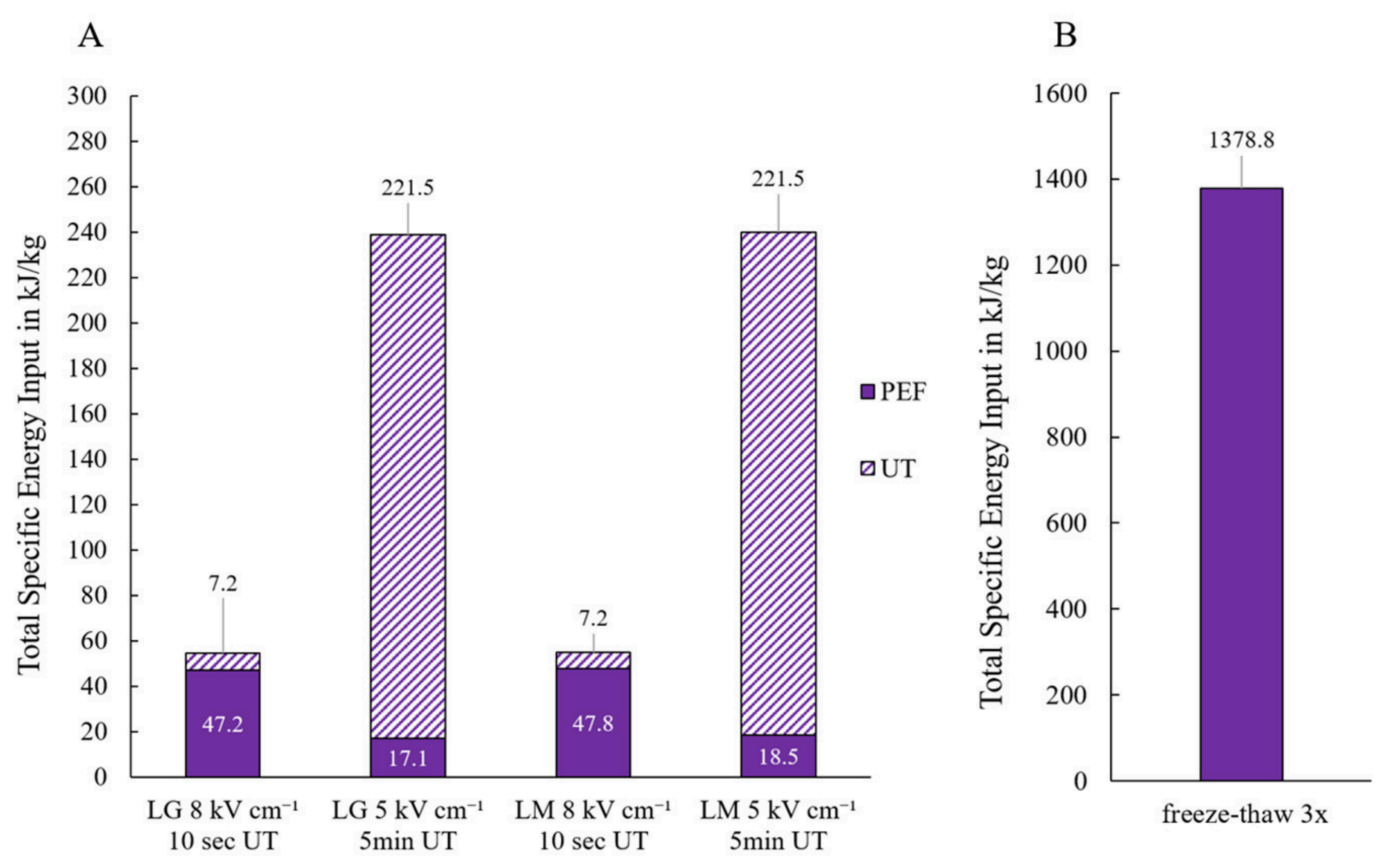
Total specific energy input of PEF-assisted extraction in combination with homogenization (**A**) and conventional freeze–thaw process (**B**).

According to Kronbauer, M. et al. 2023 [25], optimal protein yields from milled nettle leaves were obtained at comparatively low specific energy inputs of approximately 20 kJ/kg. Conversely, Yu, X. et al., 2015 [57] demonstrated that markedly higher energy inputs, around 160 kJ/kg, were necessary to achieve a pronounced enhancement in protein recovery from rapeseed leaves, which has been primarily related to the plant cell structures, mentioned in 3.1. Moreover, this study determined the total energy requirements of the whole extraction process. Therefore, the duration of homogenization was additionally taken into account for process evaluation. Furthermore, conventional freezing and thawing were evaluated as a benchmark for traditional cell disruption and protein release, providing a basis for comparison to PEF treatment in terms of energy input and extraction performance. Considering the two different homogenization durations used, a treatment time of 10 s consumed 7.2 kJ/kg, whereas an extended homogenization of 5 min required 221.54 kJ/kg (see Supplementary Materials S4). In combination, homogenization of 10 s and PEF treatment, using 8 kV cm^−1^, resulted in a total specific energy input of 54.4 kJ/kg for LG and 55.0 kJ/kg for LM. In contrast, the combination of 5 min of homogenization and subsequent PEF treatment at 5 kV cm^−1^ yielded total energy inputs of 238.6 kJ/kg for LG and 240.0 kJ/kg for LM (Figure 5).

Among all PEF treatments, the combination of 5 kV cm^−1^ with 5 min of homogenization resulted in the highest total process-specific energy consumption, which is related to the extended homogenization time. However, it also achieved the maximum protein extraction regarding the defined extraction processes.

In comparison, the application of an electric field strength of 8 kV cm^−1^ combined with a homogenization duration of only 10 s resulted in the second-highest protein yield, particularly for LG, while requiring substantially less total process-specific energy input, which is solely due to the significantly shorter homogenization time. However, the practical application of 8 kV cm^−1^ was constrained by reaching the system’s maximum current limit (500 A). For benchmarking, the conventional cell disruption method (freezing and thawing), was found to require a substantially higher specific energy input of 1378 kJ/kg. This demonstrates the greater efficiency of PEF-based treatments based on higher protein extraction results and energy efficiency under the investigated conditions.

### 3.3. Cell Disintegration

The conductivity measurements (left y-axis) showed a stepwise increase from the untreated suspension to homogenized samples and, subsequently, to samples subjected to combined homogenization and PEF treatment. The homogenization time was the primary factor governing cell disruption, as indicated by the increased conductivity resulting from the release of intracellular electrolytes.

**Figure 6:**
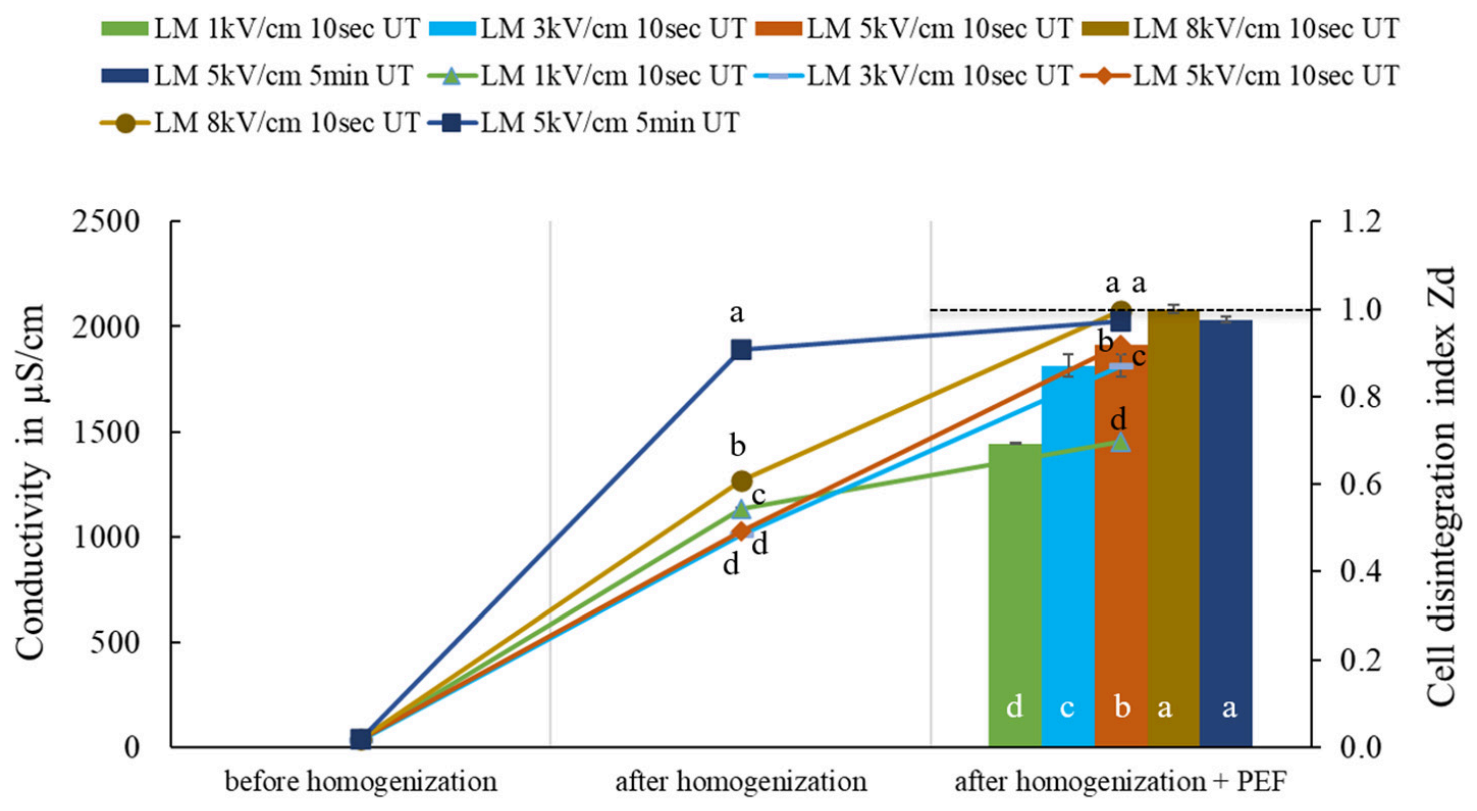
Conductivity and cell disintegration index of LM in consideration of different PEF processing with different lowercase letters showing significant difference at *p* < 0.05, according to Tukey post hoc test.

**Figure 7:**
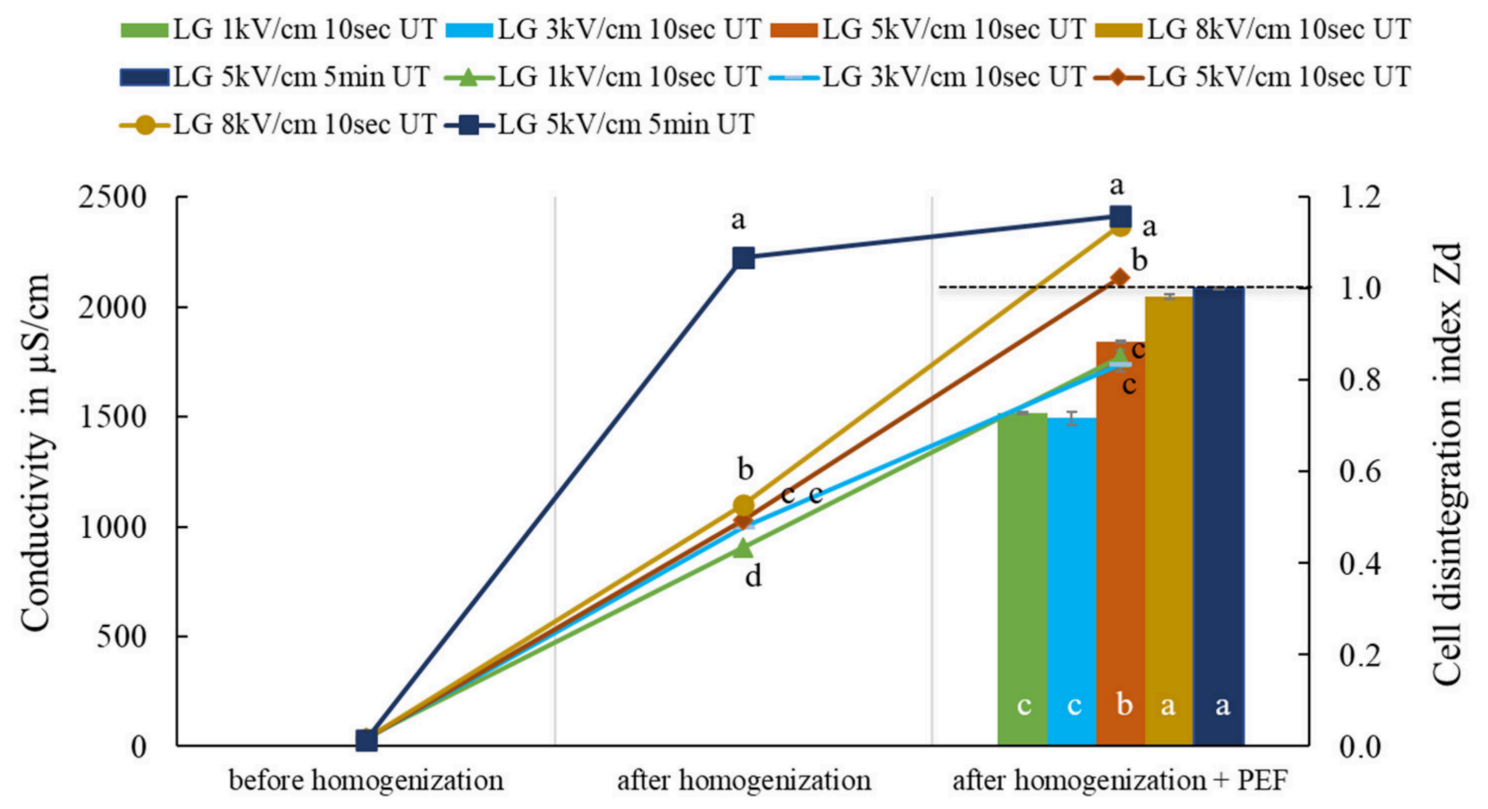
Conductivity and cell disintegration index of LG in consideration of different PEF processing with different lowercase letters showing significant difference at *p* < 0.05, according to Tukey post hoc test.

In Figure 6 and Figure 7, the electrical conductivity (left y-axis) of the duckweed suspensions before homogenization was 36 ± 3.2 µS/cm. Following 5 min of ULTRA-TURRAX homogenization, conductivity increased markedly to 2057 ± 25 µS/cm (after homogenization), indicating extensive disruption of plant tissue, cell rupture and the release of intracellular electrolytes. This increase was substantially greater than that observed after 10 s of homogenization (1059 ± 107 µS/cm), which also exhibited considerably higher variability, suggesting a less homogeneous disruption of the plant material. Subsequent application of PEF treatment after 5 min homogenization (after homogenization+PEF) resulted in a further, albeit modest, increase in conductivity to 2220 ± 212 µS/cm, indicating that PEF still contributed to additional membrane permeabilization and the release of conductive intracellular compounds. However, the influence of PEF was more pronounced in the protein and antioxidant analyses (Section 3.1, Section 3.4 and Section 3.5).

The cell disintegration index (Z_d_) enables the determination of the degree of cell membrane permeabilization or electroporation, and the associated release of intracellular substances, which has led to an increase in the conductivity of the medium. The Z_d_ evaluation is a commonly used method to illustrate the degree of cell disintegration after PEF treatment [27,58,59,60]. In the case of LM, only the highest field strength of 8 kV cm^−1^ resulted in a Z_d_ of 1 (maximum marked by black dotted line), which suggests complete cell membrane permeabilization (Figure 6, right y-axis). However, applying an electric field strength of 5 kV cm^−1^ combined with a homogenization time of 5 min resulted in a Z_d_ index close to 1 (0.98), indicating extensive membrane permeabilization and the effect of irreversible electroporation. By comparison, the reduction of homogenization time to 10 s at the same field strength led to a significantly lower Z_d_ value of 0.92, although this may still reflect pronounced electroporation effects.

The impact of electric field strength and homogenization reveals a stepwise increase in Z_d_ from 1 to 8 kV cm^−1^ followed after 10 s of homogenization as well as 5 min of homogenization prior to 5 kV cm^−1^ PEF treatment. These results are in agreement with other studies reporting a gradual rise in conductivity associated with increasing electric field intensity, pulse frequency and the resulting specific energy input [57,58,59,60].

Considering the Z_d_ values obtained following PEF treatment of LG (Figure 7), a Z_d_ index of 1 (maximum marked by black dotted line) was achieved at an electric field strength of 5 kV cm^−1^ following 5 min of homogenization. Treatments at 8 kV cm^−1^ and 5 kV cm^−1^, each preceded by 10 s of homogenization, resulted in Z_d_ values of 0.98 and 0.88, respectively. The lower *Z*d value obtained at 5 kV cm^−1^ indicates that this electric field strength was insufficient to achieve complete cell permeabilization compared with 8 kV cm^−1^. The results demonstrate that membrane permeabilization of both *Lemna* species was efficient and comparable when applying either 8 kV cm^−1^ after short-term homogenization (10 s) or 5 kV cm^−1^ after prolonged homogenization (5 min). This observation is further supported by the protein extraction results, which revealed the highest protein yields under these treatment conditions.

### 3.4. Total Chlorophyll and Carotenoids

PEF treatment had a significant effect on pigment extraction, as evidenced by an increase in the total content of chlorophyll *a* + *b* (Chl) and carotenoids. To evaluate the specific influence of PEF technology on pigment extraction, an aqueous extraction phase was collected after PEF extraction and filtered to obtain the retentate, which was analyzed spectrophotometrically for total chlorophyll *a* + *b* and total carotenoid, as well as UV–Vis spectra, and thin-layer chromatographic profiles.

**Figure 8:**
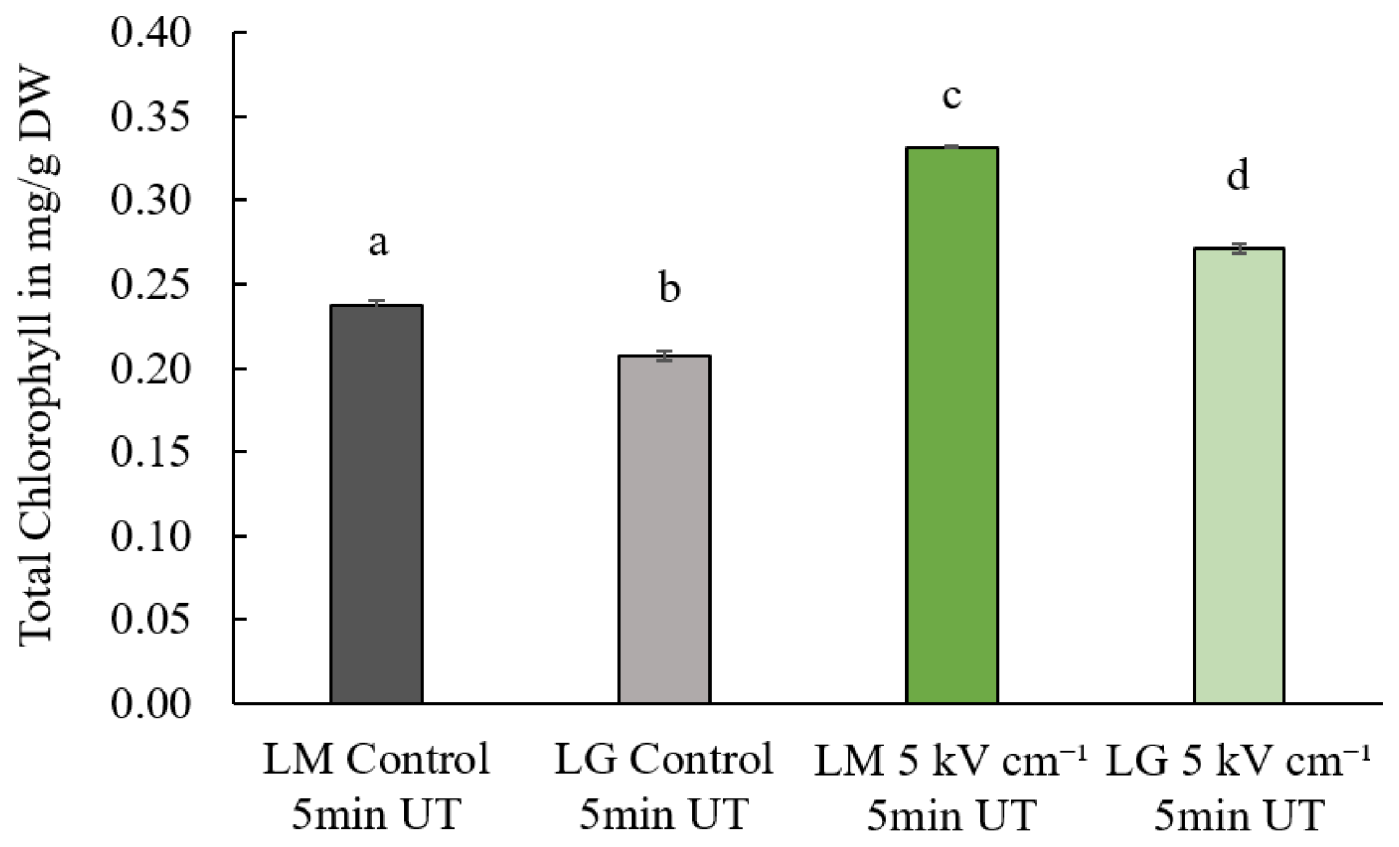
Total chlorophyll content at 5 kV cm^−1^ following 5 min homogenization with different lowercase letters showing significant difference at *p* < 0.05, according to Tukey post hoc test.

The application of PEF treatment at 5 kV cm^−1^ following homogenization increased chlorophyll (Chl) extraction in both *Lemna* species (Figure 8). Total Chl extraction reached 0.33 mg/g DW for LM and 0.27 mg/g DW for LG after PEF treatment. By comparison, the corresponding control samples, which were subjected only to 5 min of homogenization, yielded Chl extraction levels of 0.24 mg/g DW for LM and 0.21 mg/g DW for LG. These findings indicate that PEF treatment provided an additional benefit over mechanical homogenization alone.

In consideration to the total chlorophyll content for LG flour, which is 12.63 mg/g ± 0.050 DW, performed by Maag, P. et al., 2025 [2] and 12.13 mg/g ± 0.010 DW for LM flour [61], it can be stated that PEF-assisted extraction alone released approximately 0.3 mg/g DW of total Chl from homogenized fresh duckweed into an aqueous medium. Comparable results have been obtained by PEF-assisted aqueous extraction of microalgae *Chlorella pyrenoidosa*, reaching total Chl extraction values of 0–0.6 mg/g DW at 3 kV cm^−1^, 44 pulses and 99 kJ/kg [36]. Notably, the energy input used in that study for algal treatment was considerably higher (99 kJ/kg), whereas similar total chlorophyll extraction yields were obtained in the present study at lower energy inputs of 17.1 and 18.5 kJ/kg. However, it was emphasized that hydrophobic solvent, such as DMSO, used in their study, significantly increased the extraction with PEF, as chlorophyll and carotenoids are hydrophobic compounds [36].

Furthermore, incubation in the range of 0–120 min after PEF treatment, similar to the protein extraction results in Section 3.1, could also not be identified as a relevant factor influencing Chl or carotenoid release from both LM and LG (see Supplementary Materials S5, Figures S4, S5, S7 and S8). Moreover, pulse type variation (unipolar, bipolar) resulted in significant differences at lower electric field strengths (1 and 3 kV cm^−1^) in LM, where unipolar pulses enhanced chlorophyll extraction more effectively than bipolar pulses, whereas no such effect was observed for LG. This pattern was not observed for carotenoids. At higher electric field strengths, particularly at 5 kV cm^−1^, bipolar pulses tended to promote greater chlorophyll and carotenoid release (see Supplementary Materials S5, Figures S6 and S9).

**Figure 9:**
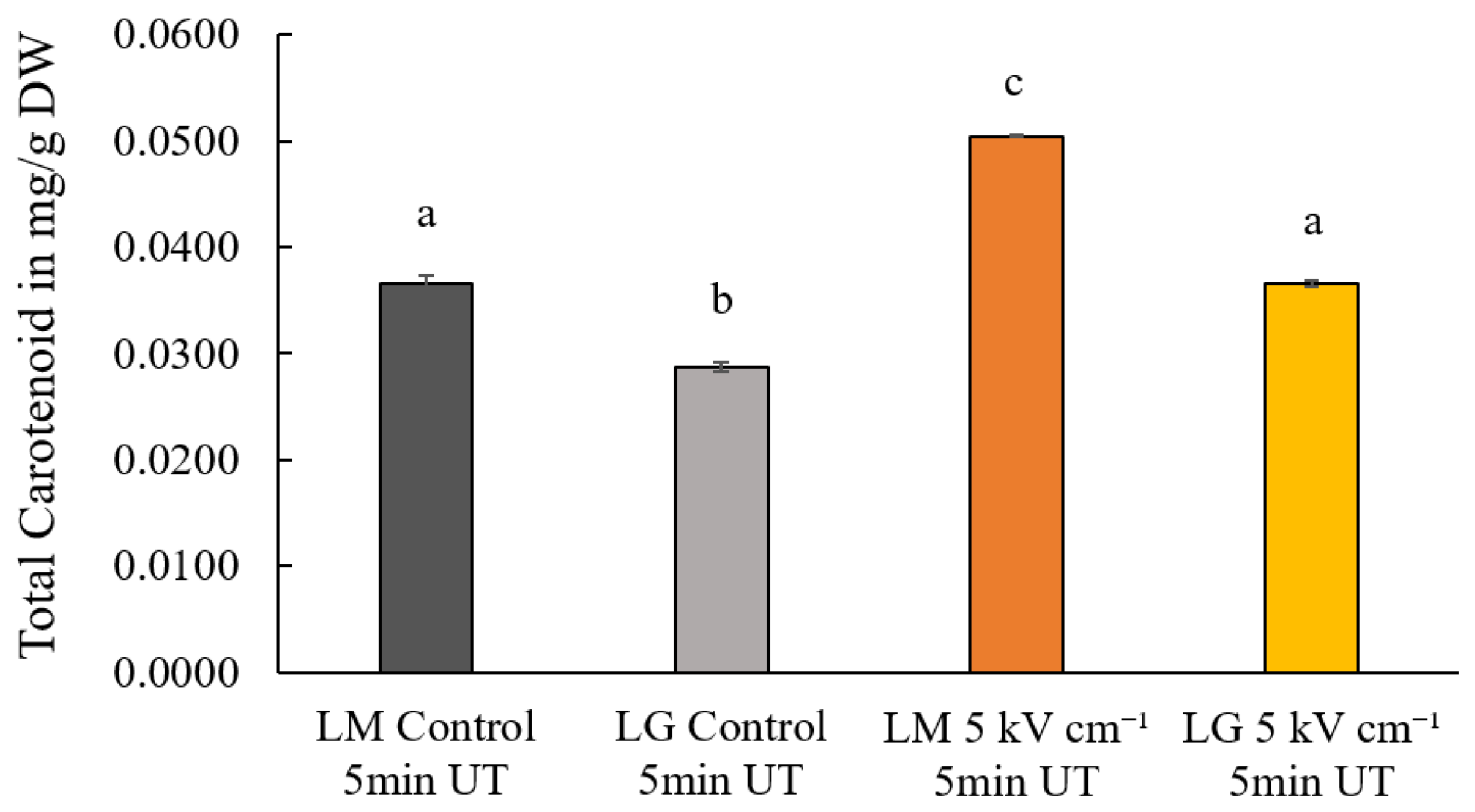
Total carotenoid content at 5 kV cm^−1^ following 5 min homogenization with different lowercase letters showing significant difference at *p* < 0.05, according to Tukey post hoc test.

Regarding the PEF-assisted extraction of total carotenoids, similar patterns have been examined, comparable to those observed for chlorophyll extraction. As shown in Figure 9, PEF exhibited a substantial increase in carotenoid extraction yield, starting with 0.0287 mg/g DW for LG and 0.0366 mg/g DW for LM in the control samples and reaching 0.0366 mg/g DW for LG and 0.0505 mg/g DW for LM after PEF treatment.

#### 3.4.1. UV–Vis Spectra of Total Carotenoids and Chlorophyll

**Figure 10:**
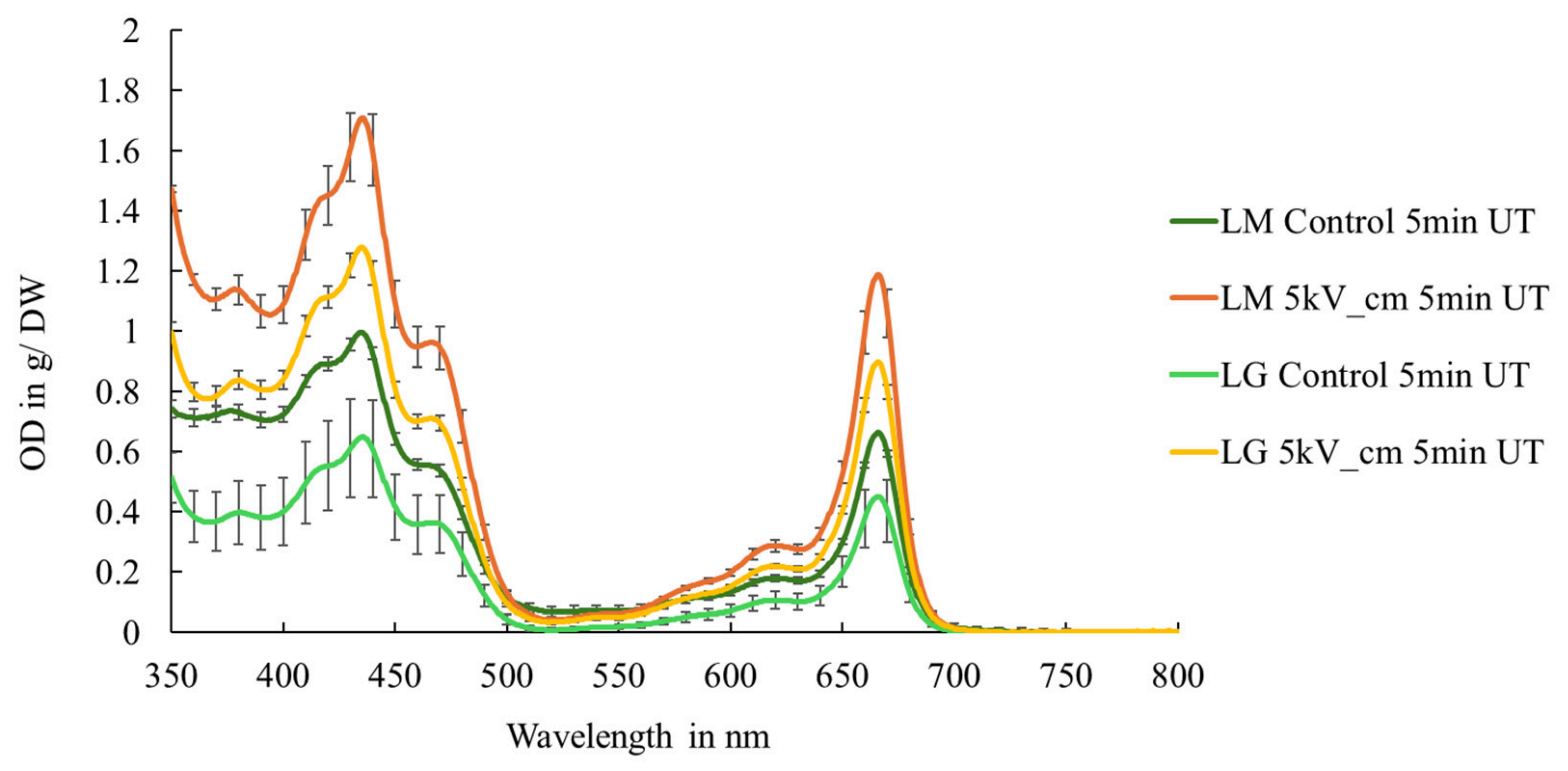
UV–Vis spectrogram of PEF-assisted extraction at 5 kV cm^−1^ following 5 min homogenization.

The UV–Vis Spectra confirms that PEF-assisted extraction significantly increases the pigment yield for both *Lemna* ssp. compared to the control, which is in line with the results of Section 3.4, Figure 8 and Figure 9. Each UV–Vis spectrum (Figure 10) exhibited a distinct peak at 665 nm, corresponding to chlorophyll a, and chlorophyll b that appears usually at 649 nm is only weakly present, as proven in Section 3.4.2. Carotenoids such as neoxanthin, violaxanthin, antheraxanthin, lutein, zeaxanthin and β-carotene, which typically occur within the range of approximately 410–480 nm [62], were detected in LG and LM [63] and are reflected by distinct peaks and shoulders in the spectra. The presence of various carotenoids in other duckweed species has also been confirmed [1]. Additionally, the spectra showed a slight increase in optical density towards shorter wavelengths, indicating the possible presence of unsaturated fatty acids.

#### 3.4.2. Thin-Layer Chromatography

**Figure 11:**
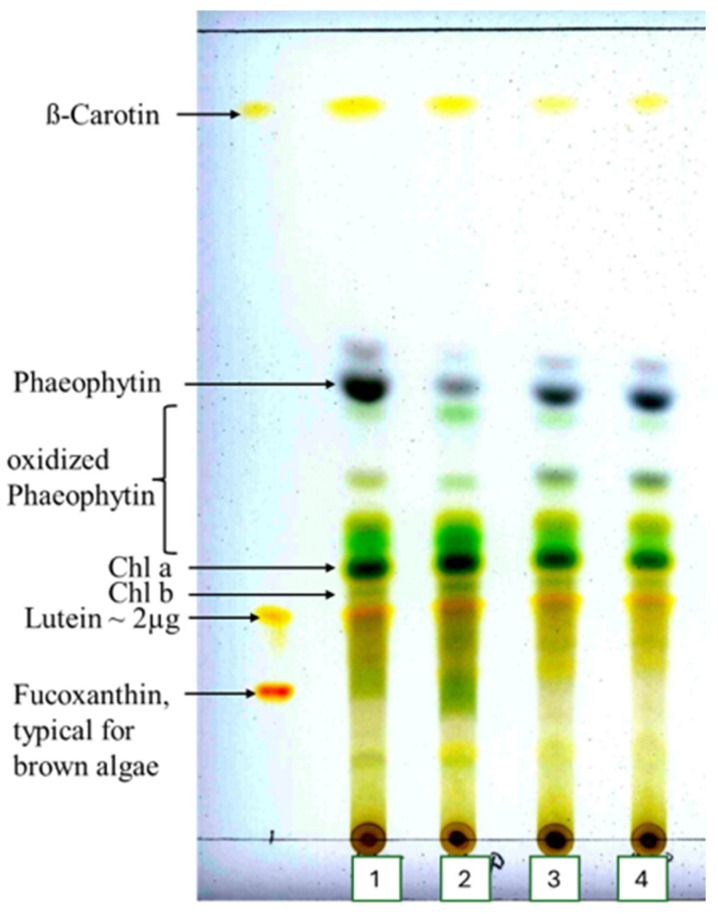
Thin-layer chromatography of LM control 5 min UT (1), LM 5 kV cm^−1^ 5 min UT (2), LG control 5 min UT (3) and LG 5 kV cm^−1^ 5 min UT (4).

Thin-layer chromatography (TLC), shown in Figure 11, clearly confirmed the presence of β-carotene and lutein based on co-applied marker pigments, the existence of which has been confirmed by the literature [1]. The appearance of lutein bands has likewise been illustrated in studies examining the lipid composition of wild-type and transgenic duckweed (*Lemna japonica*) lines [64]. Chlorophylls and pheophytins were identified based on their characteristic colors and retention factors (Rf), in accordance with established reference data. Overall, the extract obtained from LM generated a slightly more pronounced signal compared to that of LG. This finding is consistent with the results obtained for the other analytes investigated. The effect of PEF treatment is less pronounced in the TLC analysis compared to spectral analysis. However, it is conceivable that treatment with an ULTRA-TURRAX homogenizer leads to the formation of a microparticulate phase, which competes with the stationary phase of the TLC for pigment binding. This interpretation is supported by the observation that a considerable amount of material remains at the application site, accompanied by the appearance of a multiple band pattern between the point of application and the chlorophyll b band. This finding suggests either the presence of structural polymorphism within the pigment material or a delayed migration of components from the application site into the mobile phase during chromatography. Such effects could also result from overloading of the gel. However, it is more likely that co-extracted lipids interfere with the adsorption capacity of the stationary phase, thus adversely affecting the elution behavior of the analytes on the TLC plate. Notably, a distinct banding pattern can be observed between the chlorophyll a and pheophytin bands in samples treated with PEF, which may correspond to oxidized pheophytin, exhibiting increased polarity.

### 3.5. Total Polyphenols

**Figure 12:**
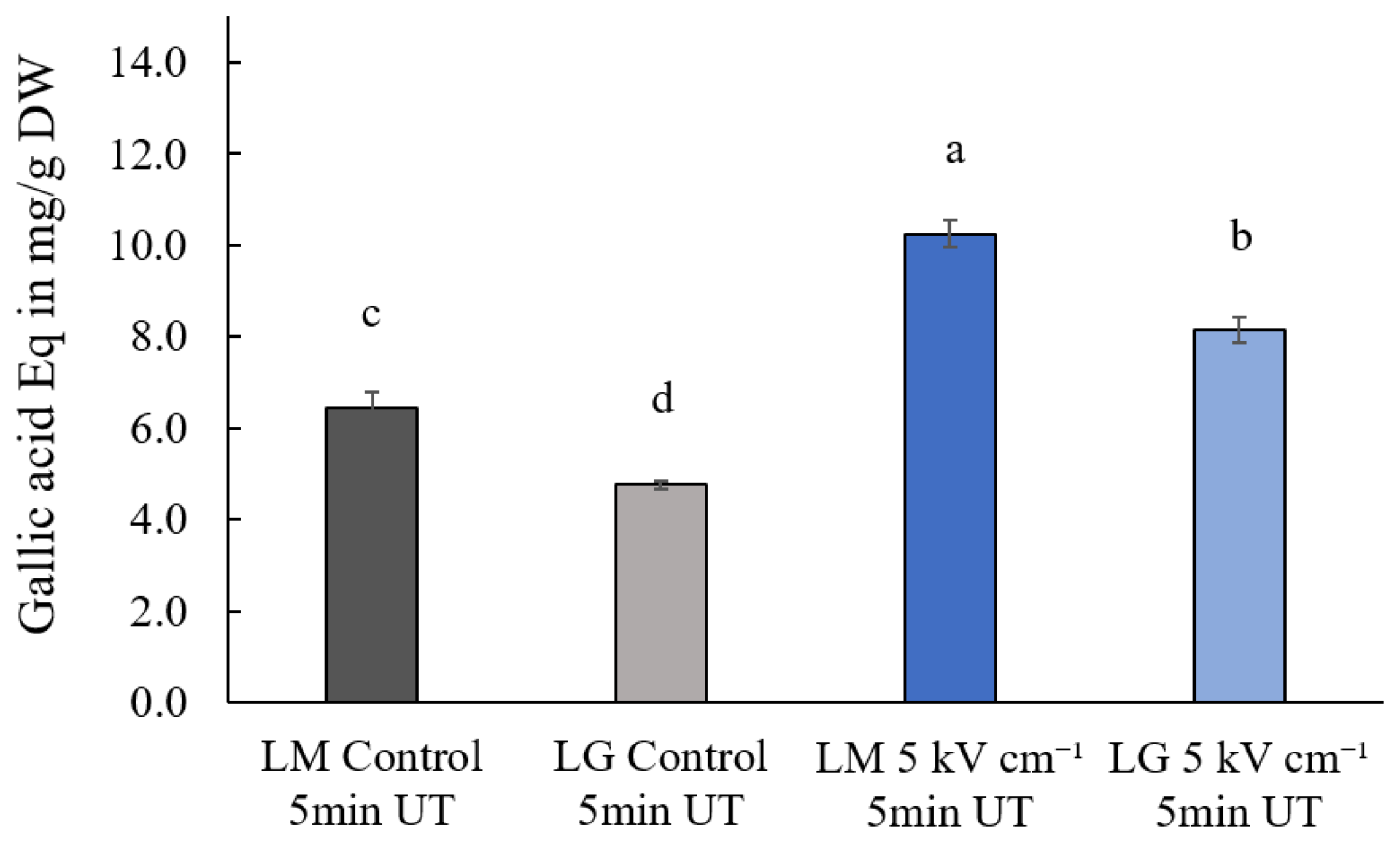
Total polyphenol content at 5 kV cm^−1^ following 5 min homogenization with different lowercase letters showing significant difference at *p* < 0.05, according to Tukey post hoc test.

Application of PEF-assisted extraction resulted in an approximately 40% increase in polyphenol recovery from suspended and homogenized duckweed (LM and LG). Specifically, LM exhibited a polyphenol extraction yield of 10.26 mg/g, significantly exceeding that of the corresponding untreated LM control sample (6.44 mg/g) after 5 min of homogenization (Figure 12). Similarly, PEF-treated LG resulted in 8.16 mg/g of extracted polyphenols, whereas the control sample achieved only 4.76 mg/g of extracted polyphenols. Moreover, incubation time after PEF extraction (0–120 min) showed no distinct effect on enhanced polyphenol release in either LM and LG (see Supplementary Materials S6, Figures S10 and S11), consistent with the observations reported in Section 3.1 and Section 3.4. Likewise, variation in pulse type (unipolar, bipolar) did not significantly influence polyphenol extraction (see Supplementary Materials S6, Figure S12).

Since no published studies on PEF-assisted polyphenol extraction from duckweed are currently available, comparisons with the existing literature remain limited [5,11]. Accordingly, the results reported from PEF-treated green leafy plants served as a basis for comparison with the corresponding results obtained for duckweed. Rapeseed leaves and stems have been treated by PEF extraction to recover proteins and polyphenols [57]. Interestingly, the highest extracted polyphenol yield was determined at an electric field strength of 20 kV cm^−1^ (>90%). However, 5 kV cm^−1^, which was lower in extraction yield, resulted in the highest polyphenol purity (91%) in aqueous solution for rapeseed leaves, emphasizing a higher selectivity in extraction, though a polyphenol extraction yield of 55% [57] was achieved which means an approximate increase of 45% to the untreated sample, similarly to the results of this study (40%). Another study examined low electric field strengths, at around 1 kV cm^−1^, to be proven effective for the extraction of polyphenols from fresh green tea leaves [65]. Low electric field strengths could also be confirmed by Jara-Quijada, E. et al., 2024a [66], who demonstrated a significant polyphenol extraction yield of 50% from green tea leaves at 5.88 kV cm^−1^ compared to conventional maceration.

Research on PEF-assisted polyphenol extraction has focused on various food sources, including fruits (e.g., strawberries, grapes—red wine), herbs (e.g., thyme, rosemary), vegetables (e.g., beet-root) and food by-products (e.g., potato peels), each of which offers its own benefits. This includes enhanced antioxidant contents in juices, improving the extraction efficiency of polyphenols from wine grapes to reduce maceration time, or increasing health-promoting properties of herbs and food by-products in the context of sustainable food utilization, which underscores the positive effects of PEF treatment [24,60,65,66,67,68,69]. Previous studies have already emphasized the potential of duckweed as a source of polyphenols, particularly flavonoids, using conventional extraction methods applied to either fresh or dried biomass [1,3,11,70,71,72]. This research provides the first evidence that polyphenols can be successfully extracted from duckweed using PEF technology. These compounds are associated with various health-promoting properties, including the prevention of chronic diseases such as cardiovascular disorders and diabetes [48,67,73]. However, in food applications, polyphenols are controversially discussed, as they may also reduce protein digestibility and contribute to undesirable dark brown discoloration.

## 4. Conclusions

This exploratory proof-of-concept study demonstrates the first application of PEF for protein extraction from *Lemna minor* and *Lemna gibba*, proving its potential as a novel and effective alternative to conventional pre-treatment methods. Different electric field strength intensities have been tested to assess their impact on protein and bioactive compound extraction, with an optimized homogenization time that significantly enhanced extraction efficiency and facilitates additional protein release upon PEF application.

Critically, PEF-assisted treatments were 5–25 times more energy efficient than conventional freeze–thaw (54–240 vs. 1378 kJ/kg), highlighting preliminary process advantages despite trade-offs between yield and energy input. *L. minor* consistently outperformed *L. gibba*, suggesting species selection for targeted applications. These first-of-kind data for *Lemna* ssp. establish PEF as a promising green technology for plant protein pre- processing and provide parameter ranges and mechanistic insights to guide future research. Further research efforts should target the integration of PEF-assisted extraction into a full-scale process for the extraction, purification and concentration of duckweed protein for powder production, given that no studies have yet examined this approach.

## Data Availability

The original contributions presented in this study are included in the article/Supplementary Material. Further inquiries can be directed to the corresponding author.

## Supplementary Materials

The following supporting information can be downloaded at https://www.mdpi.com/article/10.3390/foods15152671/s1; S1. BSA calibration curve (relevant for Section 2.3), Figure S1: BSA calibration curve; S2. Effect of pulse type and incubation time (relevant for Section 3.1), Figure S2: Protein extraction at different electric field strengths (A: 1 kV cm^−1^, B: 3 kV cm^−1^, C: 5 kV cm^−1^) from duckweed *Lemna minor* (solid line) and *Lemna gibba* (dashed line) under the influence of pulse type unipolar (blue) and bipolar (orange) and incubation time 0–120 min compared to respective non-PEF-treated control. The results were statistically analyzed using multivariate analysis of variance (MANOVA) and one-way analysis of variance (ANOVA) at a *p* < 0.05 significance level, followed by Tukey post hoc test to determine statistical significance of the data collected; S3. Homogenization time (relevant for Section 3.1), Figure S3: ULTRA-TURRAX homogenization time and rest after 8 min up to 20 min; S4. PEF Energy Input (relevant for Section 3.2); S5. Total chlorophyll a+b and total carotenoids (relevant for Section 3.4), Figure S4: Total chlorophyll a + b content of *Lemna minor* after PEF treatment following 10 s homogenization considering different incubation times, Figure S5: Total chlorophyll a + b content of *Lemna gibba* after PEF treatment following 10 s homogenization considering different incubation times, Figure S6: Total chlorophyll content at 1, 3, 5 kV cm^−1^ with unipolar and bipolar mode and 8 kV cm^−1^ at unipolar mode following 10 s of homogenization, Figure S7: Total carotenoids content of *Lemna minor* after PEF treatment following 10 s homogenization considering different incubation times, Figure S8: Total carotenoids content of *Lemna gibba* after PEF treatment following 10 s homogenization considering different incubation times, Figure S9: Total carotenoid content at 1, 3, 5 kV cm^−1^ with unipolar and bipolar mode and 8 kV cm^−1^ at unipolar mode following 10 s of homogenization; S6. Total polyphenols (relevant for Section 3.5), Figure S10: Polyphenolic content of *Lemna minor* after PEF treatment following 10 s homogenization considering different incubation times, Figure S11: Polyphenolic content of *Lemna gibba* PEF treatment following 10 s homogenization considering different incubation times, Figure S12: Polyphenol content at 1, 3, 5 kV cm^−1^ with unipolar and bipolar mode and 8 kV cm^−1^ at unipolar mode following 10 s of homogenization; S7. Folin–Ciocalteu calibration line of phenolic content (relevant for Section 2.7 and Section 3.5), Figure S13: Folin–Ciocalteu calibration curve. List of Abbreviations.
