# Supplementary material for: PEF-Assisted Extraction of Proteins and Bioactive Compounds from Duckweed (Lemna ssp.): An Exploratory Proof-of-Concept Study
Source: Foods. 2026 Jul 29;15(15):2671. doi: 10.3390/foods15152671 (PMC13464449; doi:10.3390/foods15152671)
Supplement: Supplementary file 1 [file foods-15-02671-s001.zip › foods-4426637-supplementary.pdf]

Supplementary Material

S1. BSA calibration curve (relevant for section 2.3)

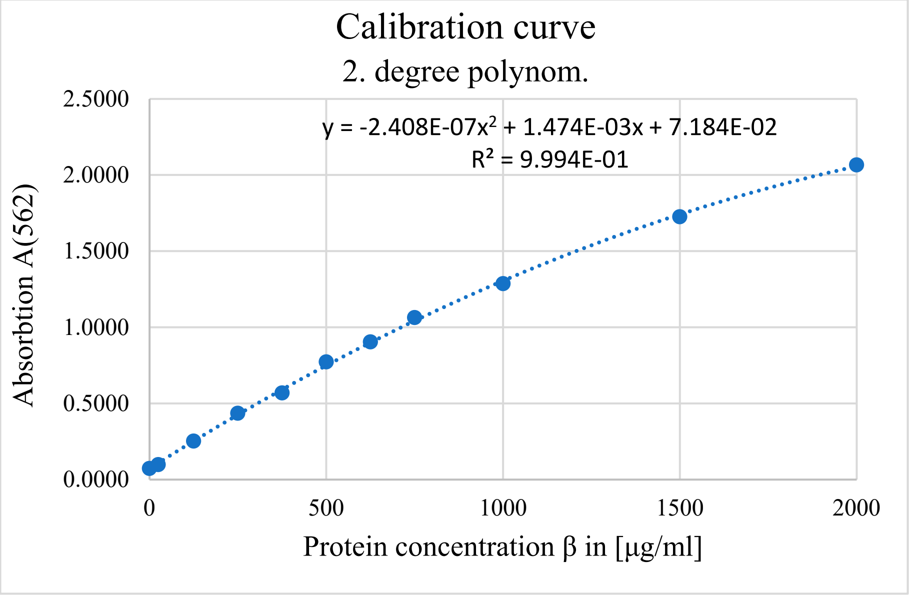

Figure S1: BSA calibration curve.

S2. Effect of Pulse Type and Incubation Time (relevant for section 3.1)

The effect of PEF-assisted protein extraction from duckweeds *Lemna minor* (LM) and *Lemna gibba* (LG) was tested under different electric field strengths (1, 3, and 5 kV cm^{-1}), pulse types (unipolar, up and bipolar, bip) and incubation times, illustrated in Figure S2 (graphs A, B and C).

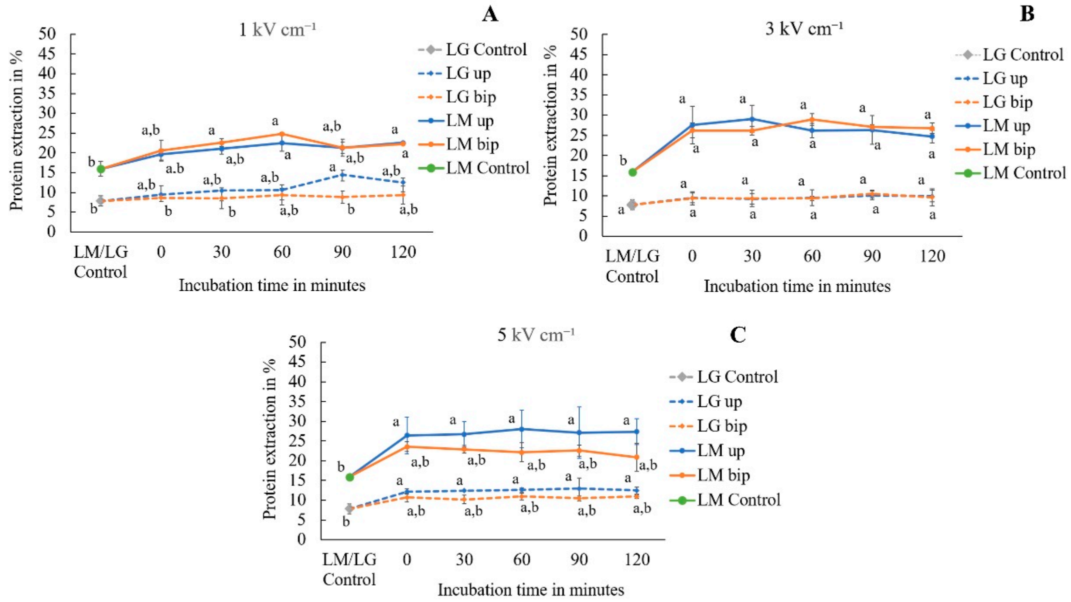

Figure S2: Protein extraction at different electric field strengths (A: 1 kV cm^{-1}, B: 3 kV cm^{-1}, C: 5 kV cm^{-1}) from duckweed *Lemna minor* (solid line) and *Lemna gibba* (dashed line) under the influence of pulse type unipolar (blue) and bipolar (orange) and incubation time 0-120 minutes compared to respective non-PEF-treated control. The results were statistically analyzed using multivariate analysis of variance (MANOVA) and one-way analysis of variance (ANOVA) at a $p < 0.05$ significance level, followed by Tukey post-hoc test to determine statistical significance of the data collected.

Prior to PEF treatment, each sample was homogenized for 10 seconds using an Ultra-Turrax homogenizer at 11,000 rpm. This pre-crushing step is essential, as PEF alone would otherwise induce only tissue softening rather than effective disintegra-

tion. With increasing electric field strength, unipolar pulses (blue line) tended to result in higher extraction efficiencies than bipolar pulses (orange line), although the differences were not statistically significant. The trend was especially apparent for LM at 5 kV cm^{-1} (see Figure S2C), whereas it was less pronounced in LG.

A similar observation was reported by Carullo, D., et al. 2020 [1], who found that bipolar pulses were less effective than unipolar pulses in extracting water-soluble compounds, including proteins, C-phycocyanin, and carbohydrates, from *Arthrospira platensis*. Moreover, in this study, the electric field strengths of 3 and 5 kV cm^{-1} showed a significant increase in protein release for LM compared to the non-PEF-treated control, while LG exhibited significantly increase only at 5 kV cm^{-1}.

For both *Lemna* species, treatment at 1 kV cm^{-1} was ineffective for protein extraction. Moreover, the incubation periods evaluated in this study did not significantly increase protein release, in contrast to the findings of Wang, M., et al. 2023 [2], who identified a 120-minute incubation time as optimal for protein extraction from *Chlorella vulgaris* using water as the extraction medium.

It can be stated that 3 and 5 kV cm^{-1} for both *Lemna ssp*. resulted in the strongest protein release with similar effectiveness ($p > 0.05$) reaching a maximum increase of protein release of around 11.2% for LM and 4.7% for LG. Overall, protein extraction from LM was found to be more promising than from LG, resulting in higher protein extraction yields. Further experiments were conducted to expand upon these findings and to elucidate the impact of homogenization on extraction efficiency in combination with PEF treatment.

S3. Homogenization Time (relevant for section 3.1)

The conductivity of the suspension increased with homogenization time until reaching a plateau after 5 min of Ultra-Turrax treatment (see Figure S3). Further extension of the homogenization period to 8 min did not enhance conductivity. In addition, no increase in conductivity was detected during a resting period of up to 20 min following the 8 min homogenization treatment, indicating that conductivity had reached its maximum value.

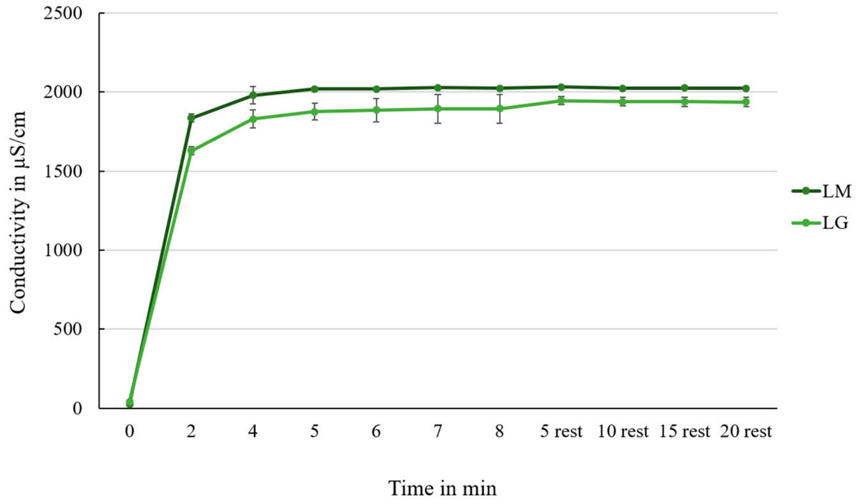

Figure S3: Ultra-turrax homogenization time and rest after 8 min up to 20 min.

S4. PEF Energy Input (relevant for section 3.2)

Energy input of homogenization by Ultra-Turrax (UT):

UT 11.000 rpm, **10 seconds** = 0.00013 kWh in kJ/kg:

0.00013 * 3600 kJ = 0.468 kJ → 0.468 kJ / 0.065 kg = 7.2 kJ/kg

UT 11.000 rpm, **5minutes** = 0.004 kWh in kJ/kg:

0.004 * 3600 kJ = 14.4 kJ → 14.4 kJ / 0.065kg = 221.54 kJ/kg

How much energy does it take to freeze 1 kg of water:

Table value from the literature [3]: 333.9 kJ (~ 334kJ)

20°C → 0°C:

Q_1 = kg * $C_{spec.}$ *T °C

Q_1 = kg * 4.18 kJ/kg * 20 = 83.6 kJ/kg

Freezing: Q_2 = 333.9 kJ

Deep-Freeze to -20°C:

Q_3 (-20) = 1*2.1*(0-(-20)) = 42 kJ

Q = Q_1 + Q_2 + Q_3 = 83.6 kJ/kg + 333.9 kJ/kg + 42 kJ/kg= 459.6 kJ/kg

3x freezing/defreezing → 459.6 kJ/kg * 3 = 1378.8 kJ/kg

S5. Total Chlorophyll a + b and Total Carotenoids (relevant for section 3.4)

The investigated incubation times did not significantly affect the release of chlorophylls accoridng to Tukey's post-hoc test ($p > 0.05$) (see Figure S4 and Figure S5). However, at an electric field strength of 5 kV cm^{-1}, a slight increase in extraction

efficiency at unipolar mode after 120 minutes incubation have been observed for both *Lemna* species but remained insignificant. The combination of electric field strength, pulse polarity, and incubation time did not reveal any consistent pattern that would indicate significant ($p > 0.05$) enhanced extraction efficiency. Therefore, the effect size was considered to provide additional statistical evidence. The eta-squared (η^2) value of 0.15 for both *Lemna* ssp. indicates the proportion of variance in the dependent variable that can be attributed to the treatment effect. In this case, 15.26% for LM and 15.23% for LG of the variance in the dependent variable is explained by differences between the treatment levels. An η^2 value of 0.15 is considered to represent a large effect size.

Furthermore, the data presented in Figure S6, which show chlorophyll extraction at electric field strengths ranging from 1 to 8 kV cm^{-1} without accounting for incubation time, revealed more distinct differences between pulse types and electric field strengths. Compared with the control, unipolar pulses significantly improved Chl extraction in LM at 1 and 3 kV cm^{-1}, whereas bipolar pulses remained ineffective. Across both *Lemna* species, the greatest Chl yields were observed at 8 kV cm^{-1} with unipolar treatment, amounting to 0.08 mg/g DW for LM and 0.05 mg/g DW for LG.

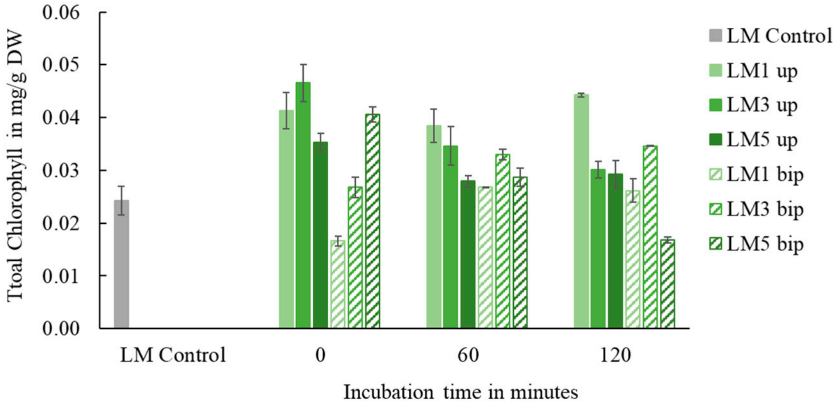

Figure S4: Total Chlorophyll a + b content of *Lemna minor* after PEF treatment following 10 seconds homogenization considerung different incubation times.

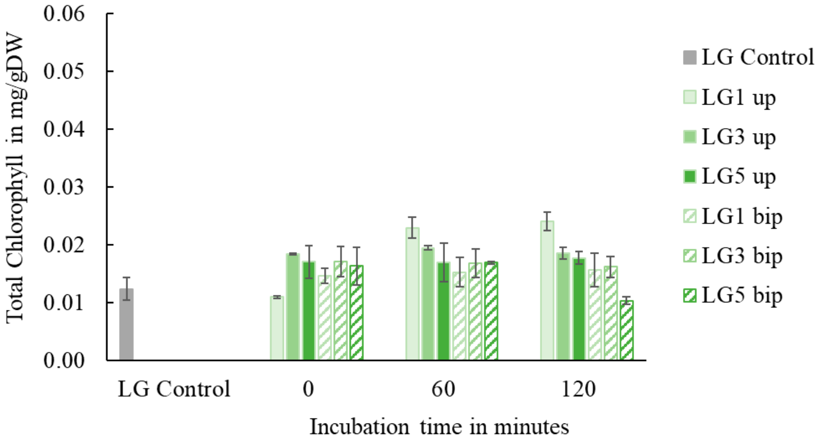

Figure S5: Total Chlorophyll a + b content of *Lemna gibba* after PEF treatment following 10 seconds homogenization considerung different incubation times.

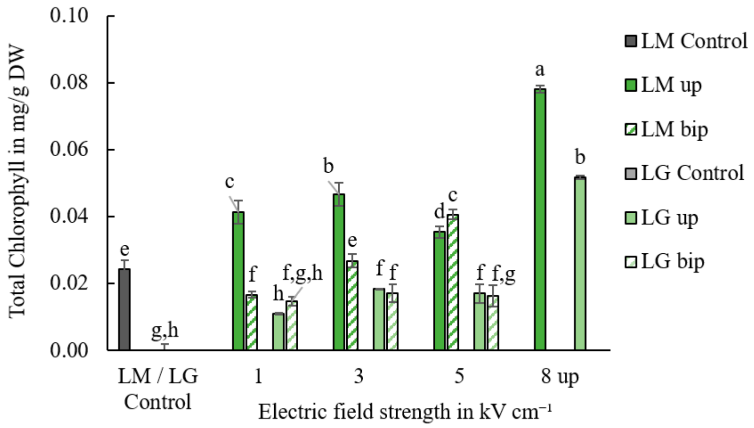

Figure S6: Total Chlorophyll content at 1, 3, 5 kV cm^{-1} with unipolar and bipolar mode and 8 kV/cm at unipolar mode following 10 seconds of homogenization.

The carotenoid extraction tended to be more efficient under unipolar pulse conditions than under bipolar conditions at electric field strengths of 1 and 3 kV cm^{-1}. However, no consistent effect of incubation time on carotenoid release was evident for both *Lemna* ssp., mirroring the observations made for chlorophyll extraction (see Figure S7 and Figure S8). Furthermore, the effect size was considered to provide additional statistical interpretation. The analysis yielded an eta-squared value of $\eta^2 = 0.81$ for LM and 0.67 for LG indicating a substantial effect of the treatment on the dependent variable. Nearly 81% for LM and 67.08% for LG of the total variance in the outcome variable can be attributed to differences between treatment conditions. Based on established guidelines for effect size interpretation, this value is classified as a large effect, highlighting the considerable practical significance of the treatment.

The application of 8 kV cm^{-1} at unipolar pulse mode even doubled the amount of extracted total carotenoids for both *Lemna* ssp., reaching 0.00157 mg/g DW and 0.0114 mg/g DW for LM and LG respectively, compared to unipolar pulses at 5 kV cm^{-1} (see Figure S9).

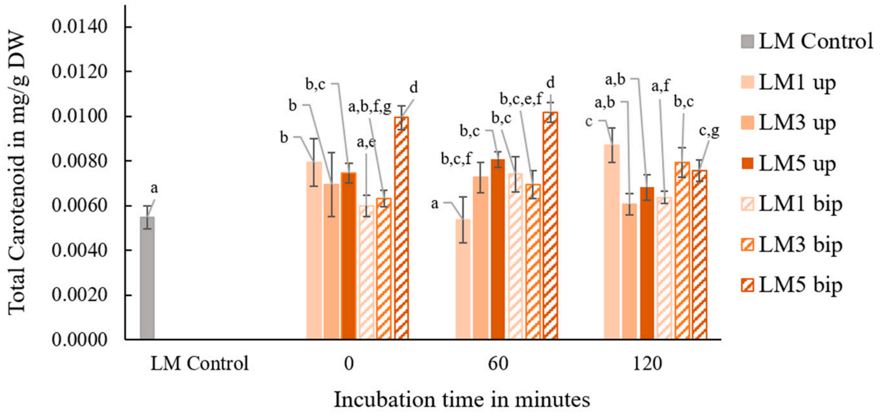

Figure S7: Total Carotenoids content of *Lemna minor* after PEF treatment following 10 seconds homogenization considerung different incubation times.

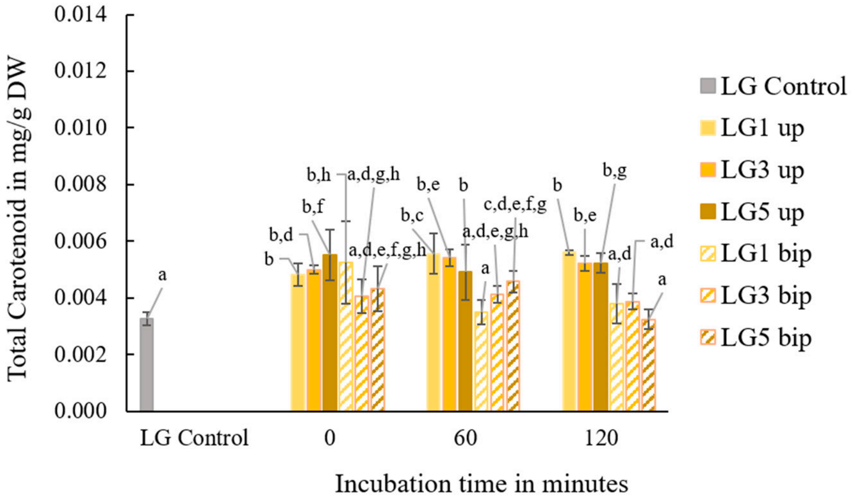

Figure S8: Total Carotenoids content of *Lemna gibba* after PEF treatment following 10 seconds homogenization considerung different incubation times.

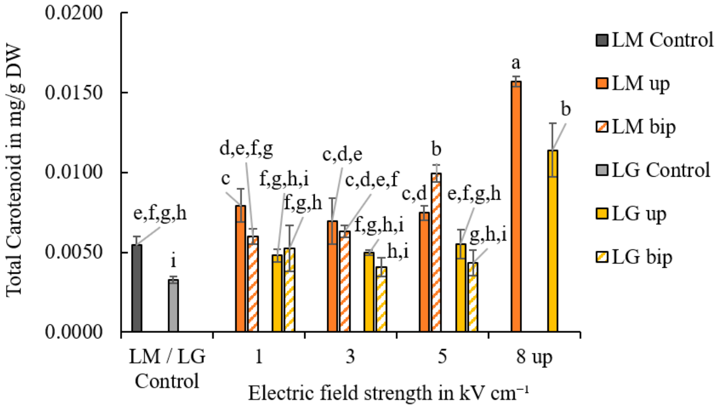

Figure S9: Total Carotenoid content at 1, 3, 5 kV cm^{-1} with unipolar and bipolar mode and 8 kV cm^{-1} at unipolar mode following 10 seconds of homogenization.

S6. Total Polyphenols (relevant for section 3.5)

PEF-assisted extraction at 3 kV cm^{-1} significantly enhanced polyphenol extraction from *Lemna* spp. under both unipolar and bipolar pulse polarities compared to the control, whereas 1 kV cm^{-1} was insufficient to improve extraction. Increasing the electric field strength to 5 kV cm^{-1} did not further enhance polyphenol recovery, as no significant differences were observed compared to 3 kV cm^{-1}. Similar to chlorophyll and carotenoids, the incubation time of 60 or 120 minutes had no strong and significant effect on the subsequent release of polyphenols in either LG or LM, with some exceptions in unipolar mode for LM (see Figure S10 and Figure S11).

Moreover, only unipolar pulses proved effective at LG from 3 kV cm^{-1} with 1.24 mg/g DW extraction yield. In contrast, application of 8 kV cm^{-1} in unipolar mode resulted in a substantial increase in polyphenol content, reaching 13.69 mg/g DW for LM and 9.74 mg/g DW for LG (see Figure S12). This is comparable to samples subjected to 5 minutes

of homogenization followed by PEF treatment at 5 kV cm^{-1} showing similar extraction yields of 10.26 mg/g DW for LM and 8.16 mg/g DW for LG.

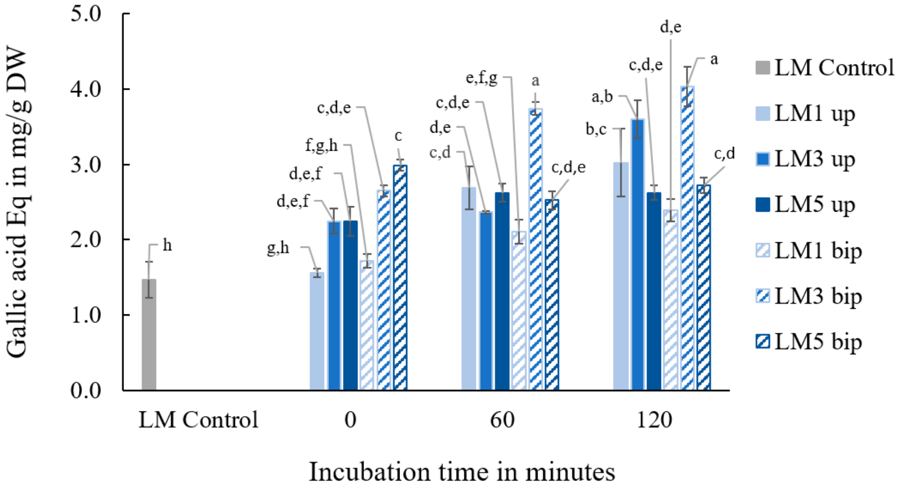

Figure S10: Polyphenolic content of *Lemna minor* after PEF treatment following 10 seconds homogenization considerung different incubation times.

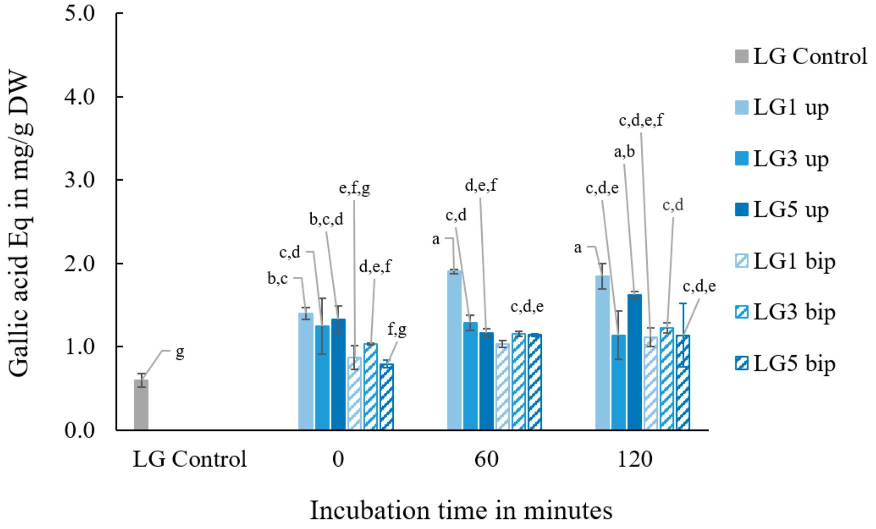

Figure S11: Polyphenolic content of *Lemna gibba* PEF treatment following 10 seconds homogenization considerung different incubation times.

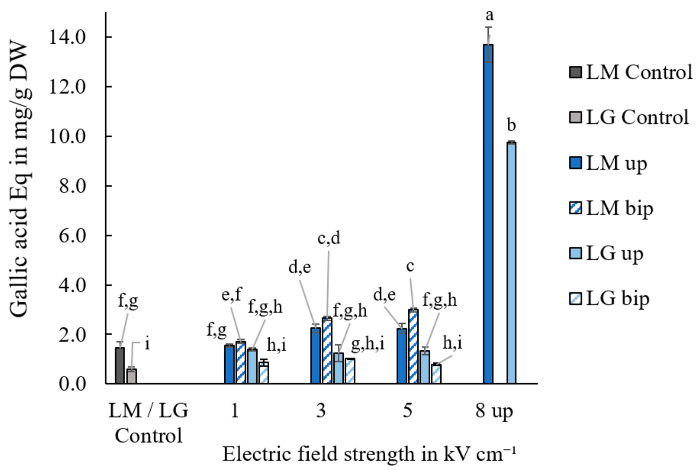

Figure S12: Polyphenol content at 1, 3, 5 kV cm^{-1} with unipolar and bipolar mode and 8 kV cm^{-1} at unipolar mode following 10 seconds of homogenization.

S7. Folin-Ciocalteu Calibration Line of Phenolic Content (relevant for section 2.7 and 3.5)

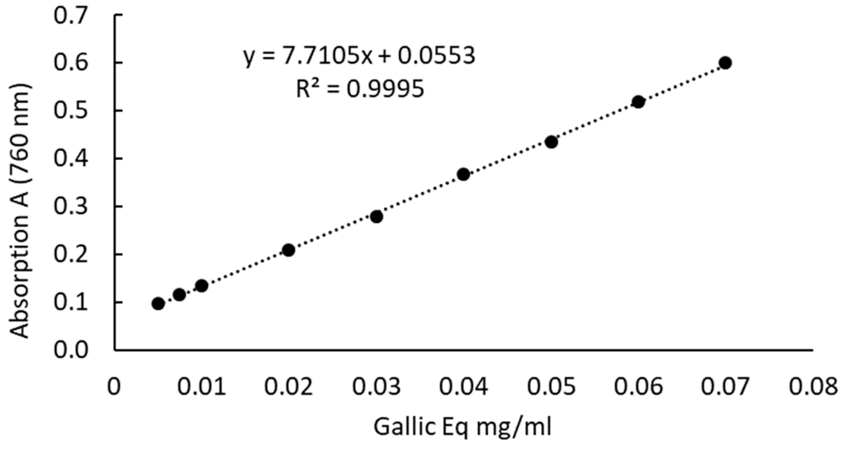

Figure S13: Folin-Ciocalteu calibration curve.

List of Abbreviations

LM	*Lemna minor*
LG	*Lemna gibba*
US	Ultrasound
UAE	Ultrasound-assisted extraction
PEF	Pulsed electric fields
PAE	PEF-assisted extraction
Up	unipolar
Bip	bipolar
Chl	Chlorophyll
GAE	Gallic acid equivalent
TLC	Thin-Layer Chromatography
BCA (Protein) Assay	Bicinchoninic acid assay
BSA	Bovine-serum albumin
min	minute(s)
sec	second(s)
g	gramm
kg	kilogramm
J	Joule
kJ	kilo Joule

References

1. Carullo, D.; Pataro, G.; Donsì, F.; Ferrari, G. Pulsed Electric Fields-Assisted Extraction of Valuable Compounds From Arthrospira Platensis: Effect of Pulse Polarity and Mild Heating. *Front. Bioeng. Biotechnol.* **2020**, *8*, 551272, doi:10.3389/fbioe.2020.551272.
2. Wang, M.; Zhou, J.; Castagnini, J.M.; Berrada, H.; Barba, F.J. Pulsed electric field (PEF) recovery of biomolecules from Chlorella: Extract efficiency, nutrient relative value, and algae morphology analysis. *Food Chem.* **2023**, *404*, 134615, doi:10.1016/j.foodchem.2022.134615.
3. Klaus Langeheinecke; Peter Hahn; Eugen Sapper. Thermodynamik für Ingenieure: Ein Lehr- und Arbeitsbuch für das Studium **2003**, doi:10.1007/978-3-322-94277-7.
